# Prediction and optimization method for welding quality of components in ship construction

**DOI:** 10.1038/s41598-024-59490-w

**Published:** 2024-04-23

**Authors:** Jinfeng Liu, Yifa Cheng, Xuwen Jing, Xiaojun Liu, Yu Chen

**Affiliations:** 1 Jiangsu University of Science and Technology, Zhenjiang, 212100 Jiangsu China; 2 Southeast University, Nanjing, 211189 China

**Keywords:** Quality prediction, Components welding, Welding quality, Process parameter optimization, Mechanical engineering, Software

## Abstract

Welding process, as one of the crucial industrial technologies in ship construction, accounts for approximately 70% of the workload and costs account for approximately 40% of the total cost. The existing welding quality prediction methods have hypothetical premises and subjective factors, which cannot meet the dynamic control requirements of intelligent welding for processing quality. Aiming at the low efficiency of quality prediction problems poor timeliness and unpredictability of quality control in ship assembly-welding process, a data and model driven welding quality prediction method is proposed. Firstly, the influence factors of welding quality are analyzed and the correlation mechanism between process parameters and quality is determined. According to the analysis results, a stable and reliable data collection architecture is established. The elements of welding process monitoring are also determined based on the feature dimensionality reduction method. To improve the accuracy of welding quality prediction, the prediction model is constructed by fusing the adaptive simulated annealing, the particle swarm optimization, and the back propagation neural network algorithms. Finally, the effectiveness of the prediction method is verified through 74 sets of plate welding experiments, the prediction accuracy reaches over 90%.

## Introduction

The shipbuilding industry is a comprehensive national high-end equipment manufacturing industry that supports the shipping industry, marine development, and national defense construction. It plays a critical role in guaranteeing national defense strength and economic development^1,2^. With the continuous development of a new generation of information technology based on big data, internet of things (IoT), 5G, cloud computing, artificial intelligence, and digital twin, the intelligent construction is becoming the dominant advanced mode in the shipbuilding industry. At the same time, welding quality control is regarded as a significant part in shipbuilding, and the related innovation research under intelligent welding also urgently needs to be carry out. The current welding processing gradually becoming more flexible and complicated, the welding quality of each workstation ultimately determines the majority of the product quality by means of propagation, accumulation and interaction.

Welding process is one of the vital industrial technologies in ship segment construction^3,4^. However, in the welding process of ship components, the local uneven heating and local uncoordinated plastic strain of metal materials are most probably leading to large residual stresses^5,6^. This will cause a reduction in the static load capacity and fatigue strength of the ship components. which in turn affects the load capacity, dimensional accuracy, and assembly accuracy of the structure. However, in most shipbuilding enterprises, quality management usually involves issuing quality plans, post-sequence production inspections, and quality statistical reports, which are static quality control. The existing welding quality prediction methods have hypothetical premises and subjective factors, which cannot meet the dynamic control requirements of intelligent welding for processing quality. These methods often encounter problems such as inefficient quality inspection, untimely quality feedback, and untimely quality control^7^. Moreover, the post-welding correction process delays the ship construction cycle and increases the production cost.

The inadequacy of traditional welding quality control technology determines the functional and technical limitations in practical applications^8,9^. Firstly, the current welding process design relies on production experience and empirical calculation formulas^10^, which makes it difficult to ensure the design requirements for minimizing residual stress in the forming of structural parts. Secondly, the absence of effective data pre-processing methods to address complex production conditions and massive amounts of welding measurement data. Currently, the welding quality prediction methods for ship components is inadequate. For example, it is difficult to balance the prediction accuracy and computational efficiency, or combine actual measured welding data to drive data analysis services.

This work aims to provide a solution to the inefficiency of welding quality control during ship construction, delaying the production cycle and increasing production costs. The proposed method has the following advantages.

- The data-acquisition framework for welding process parameters of ship unit-assembly welding is constructed, a stable and reliable data acquisition method is proposed.
- Based on the feature selection method, the influence feature of the welding quality is quantitatively analyzed. This leads to the construction of an optimal subset of process influencing features for welding quality prediction.
- Fusing an adaptive simulated annealing (SA), the particle swarm optimization (PSO) and the back propagation neural network (BPNN), a welding quality prediction model is established for welding quality control and decision making.

The remainder of this paper is organized as follows. “Related works” section presents the related research on the influence factor and prediction methods of welding quality. The data acquisition and processing framework is explained in “Acquisition and pre-processing of welding process data” section. In “Construction the welding quality prediction model” section, fusing an Adaptive SA, the PSO and the BPNN (APB), a welding quality prediction model is established. To verify the proposed method, the case study of ship unit-assembly welding is illustrated in “Case study” section. The conclusion and future work are shown in “Conclusion and future works” section.

## Related works

### Method for selecting welding quality features

For huge amount of data in the production site, the knowledge information can be mined through suitable processing methods to assist production^11,12^. Feature selection is an important and widely used technique in this field. The purpose of feature selection is to select a small subset of features from the original dataset based on certain evaluation criteria, which usually yields better performance, such as higher classification accuracy, lower computational cost and better model interpretability. As a practical method, feature selection has been widely used in many fields^13–15^.

Depending on how the evaluation is performed, feature selection methods can be distinguished as filter models, wrapper models, or hybrid models. Filter models evaluate and select a subset of features based on the general characteristics of the data without involving any learning model. On the other hand, the wrapper model uses a learning algorithm set in advance and uses its performance as an evaluation criterion. Compared to filter models, wrapper models are more accurate but computationally more expensive. Hybrid models use different evaluation criteria at different stages and combine the advantages of the first two methods.

Two versions of an ant colony optimization-based feature selection algorithm was proposed by Warren et al.^16^, which can effectively improve weld defect detection accuracy and weld defect type classification accuracy. An enhanced feature selection method combining the Relief-F algorithm with a convolutional neural network (CNN) was proposed by Jiang et al.^17^ to improve the recognition accuracy of welding defect identification in the manufacturing process of large equipment. A hybrid fisher-based filter and wrapper-based feature selection algorithm was proposed by Zhang et al.^18^, which reduces the 41 feature parameters for weld defect monitoring during tungsten arc welding of aluminum alloy to 19. The computational effort is reduced and the modeling accuracy is improved. Abdel et al.^19^ proposed a combination of two-phase mutation and gray wolf optimization algorithm in the literature to solve the wrapper-based feature selection problem. It was able to balance accuracy and efficiency in handling the classification task. To the effect of maintaining and improving the classification accuracy. Le et al.^20^ introduced a stochastic privacy preserving machine learning algorithm. The Relief-F algorithm was used for feature selection and Random Forest is utilized for privacy preserving classification. The algorithm is prevented from overfitting and higher classification accuracy is obtained.

In general, a huge amount of measured welding data is generated during the welding of actual ship components because of the complex production conditions. Problems such as high computational cost, easy to fall into local optimum and premature convergence of the algorithm may exist in feature selection. To determine the essential welding process influencing factors. It is necessary to use a suitable feature selection method that facilitates reasonable parsimony in obtaining the best set of input data features. This maximizes the accuracy and computational efficiency while reducing the computational complexity of the prediction model.

### Welding quality prediction method

As the new-generation of information technology becomes popular in the ship construction, process data from the manufacturing site can be collected. This contains a non-linear mapping relationship between welding process parameters and quality. Welding data monitoring, welding quality prediction and optimization decisions can be effectively implemented. Therefore, based on machine learning algorithms to achieve welding quality prediction has received wide attention from academia and industry.

Pal et al. predicted the welding quality by processing the current and voltage signals in the welding process^21,22^. Taking the process parameters and statistical parameters of the arc signal as input variables, the BPNN, and radial basis function network models are adopted to realize the prediction of welding quality. A Fatigue strength prediction method of ultra-high strength steel butt-welded joints was proposed by Nykanen^23^. A reinforcement-penetration collaborative prediction network model based on deep residual was designed by Lu et al.^24^ to predict reinforcement and penetration depth quantitatively. A nugget quality prediction method of resistance spot welding on aluminum alloy based on structure-borne acoustic emission signals was proposed by Luo et al.^25^.

Along with the maturity of related theories such as machine learning and neural networks, the task of welding quality prediction is increasingly implemented by scholars using related techniques.

1. Artificial neural networks (ANN): In the automatic gas metal arc welding processes, the response surface methodology and ANN models are adopted by Shim et al.^26^ predict the best welding parameters for a given weld bead geometry. Lei et al.^27^ used a genetic algorithm to optimize the initialization weights and biases of the neural network. They proposed a multi-information fusion neural network to predict the geometric characteristics of the weld by combining the welding parameters and the morphological characteristics of the molten pool. Chaki et al.^28^ proposed an integrated prediction model of ANN and non-dominated sorting genetic algorithm which was used to predict and optimize the quality characteristics during pulsed Nd:YAG laser cutting of aluminum alloys. The improved regression network was adopted by Wang et al.^29^ and predicted the future molten pool image. CNN were used by Hartl et al.^30^ to analyze process data in friction stir welding and predict the resulting quality of the weld surface. To predict the penetration of fillet welds, a penetration quality prediction of asymmetrical fillet root welding based on an optimized BPNN was proposed by Chang et al.^31^. A CNN-based back bead prediction model was proposed by Jin et al.^32^. The image data of the current welding change is acquired, and the CNN model is used to realize the welding shape prediction of the gas metal arc welding. Hu et al.^33^ established an ANN optimized by a pigeon inspired algorithm to optimize the welding process parameters of ultrasonic-static shoulder-assisted stir friction welding (U-SSFSW), which led to a significant improvement in the tensile strength of the joints. Cruz et al.^34^ presented a procedure for yielding a near-optimal ensemble of CNNs through an efficient search strategy based on an evolutionary algorithm. Able to weigh the predictive accuracy of forecasting models against calculated costs under actual production conditions on the shop floor.
2. Support vector machines (SVM): SVM using the radial kernel, boosting, and random forest techniques were adopted by Pereda et al.^35^ The direct quality prediction in the resistance spot welding process is achieved. To improve the prediction ability to welding quality during high-power disk laser welding, the SVM model was adopted by Wang et al.^36^ to predict the welding quality of the metal vapor plume. By collecting the real-time torque signal of the friction stirs welding process, Das et al.^37^ used an SVM regression model to predict the ultimate tensile strength of the welded joint. A model of laser welding quality prediction based on different input parameters was established by Petkovic^38^. Yu et al.^39^ proposed a real-time prediction method of welding penetration mode and depth based on two-dimensional visual characteristics of the weld pool.
3. Other prediction models: A neuro-fuzzy model for the prediction and classification of the defects in the fused zone was built by Casalino et al.^40^. Using laser beam welding process parameters as input variables, neural networks, and C-Means fuzzy clustering algorithms are used to classify and predict the welding defects of Ti6Al4V alloy. Rout et al.^41^ proposed a hybrid method based on fuzzy regression of the particle swarm optimization to achieve and optimize the prediction of weld quality from both mechanical properties and weld geometry. Kim et al.^42^ proposed a semantic resistance spot welding weldability prediction framework. The framework constructs a shareable weldability knowledge database based on the regression rules. A decision tree algorithm and regression tree are used to extract decision rules, and the nugget width of the case was successfully predicted. AbuShanab et al.^43^ proposed a stochastic vector functional link prediction model optimized by the Hunger Games search algorithm to link the joint properties with the welding variables, introducing a new prediction model for stir friction welding of dissimilar polymer materials.

Scholars have given various feasible forecasting schemes for welding quality. However, there are still defects, such as the lack of generalization performance of the weld quality prediction algorithms, having a large number of assumptions and subjective factors, these methods cannot meet the dynamic control requirements of intelligent welding for processing quality. Secondly, most prediction models can only predict before or after work, and cannot meet the dynamic changes in the welding environment on site. Therefore, the crucial to improving the welding quality is to give accurately and timely prediction results.

## Acquisition and pre-processing of welding process data

**Figure 1:**
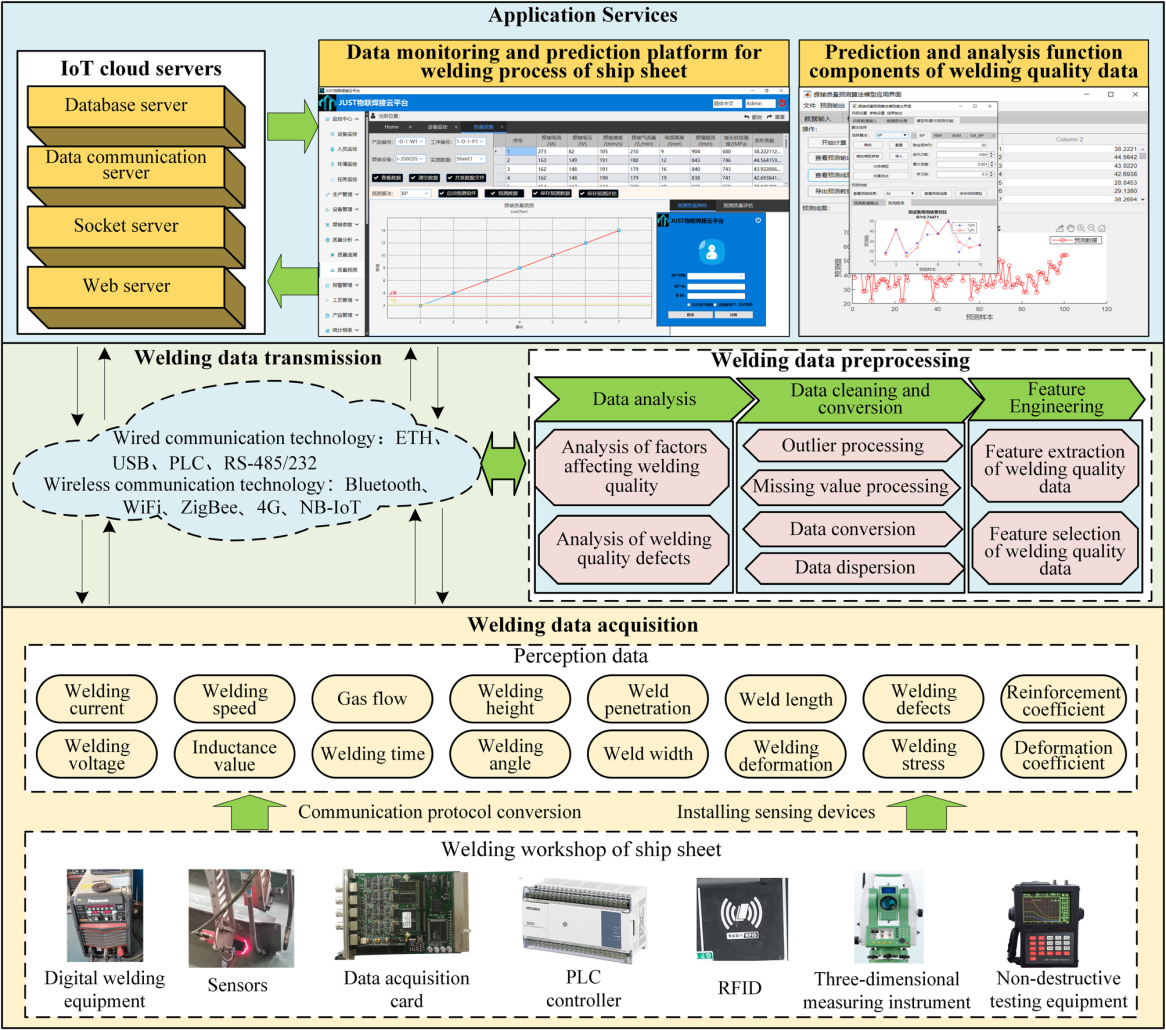
The framework of welding quality prediction method.

The welding quality prediction framework is proposed and shown in Fig. 1 (The clearer version is shown in Supplementary Figure S1). Firstly, the critical controllable quality indicators in the ship unit-assembly welding are determined, and the influencing factors are analyzed. Secondly, based on the IoT system, a data acquisition system for real-time monitoring and prediction of the welding quality of the ship unit-assembly welding is established. Collection and transmission of welding data are achieved. Then, a feature selection method is created to optimally select the key features of the welding quality data.Secondly, fusing an adaptive simulated annealing, the particle swarm optimization and the back propagation neural network, a welding quality prediction model is established for welding quality control and decision making. Finally, the welding experiments of ship unit-assembly welding as an example to verify the critical technologies in the paper.

### Analyze the factors affecting welding quality

The reasons that lead to the welding quality problems of the ship component of ships involve six significant factors: human factors, welding equipment, materials, welding process, measurement system, and production environment. Residual stresses caused by instability during the welding of ship components are inextricably linked to the welding method and process parameters used. However, the essential factors are mainly determined by the thermal welding process and the constrained conditions of the weldment during the welding process. The influencing factors of welding quality in the thermal welding process are mainly reflected in the welding heat source type and its power density $W$, the effective thermal efficiency $P$ and linear energy $Q$ of the welding process, the heat energy’s transfer method (such as heat conduction, convection, etc.) and the welding temperature field. The determinants of the welding temperature field include the nature of the heat source and welding parameters (such as welding current, arc voltage, gas flow, inductance, welding speed, heat input, etc.). When the arc voltage and welding current increase, the heat energy input to the weld and the melting amount of the welding wire will increase directly, which will affect the width and penetration of the weld. When the welding speed is too low, it will cause the heat concentration and the width of the molten pool to increase, resulting in defects such as burn-through and dimensional deformation. The restraint conditions refer to the restraint type and restraint degree of the welded structure. Its value is mainly determined by factors such as the structure of the welded sheet, the position of the weld, the welding direction and sequence, the shrinkage of other parts during the cooling process, and the tightness of the clamped part.

Take the CO2 gas shielded welding process of the ship component as an example. Welding parameters determine the energy input to the weld and to a large extent affect the formation of the weld. Important process parameters that determine the welding quality of thin plate structures of ships include arc voltage, welding current, welding speed, inductance and gas flow. For example, when the welding current is too large, the weld width, penetration, and reinforcement that determine the dimension of the weld will increase, and welding defects are likely to occur during the welding process. At the same time, the angular deformation and bending deflection deformation of the welded sheet also increase. The instability and disturbance of the melt pool and arc can be caused when the gas flow rate is too high, resulting in turbulence and spatter in the melt pool.

### Obtain the welding process data

**Figure 2:**
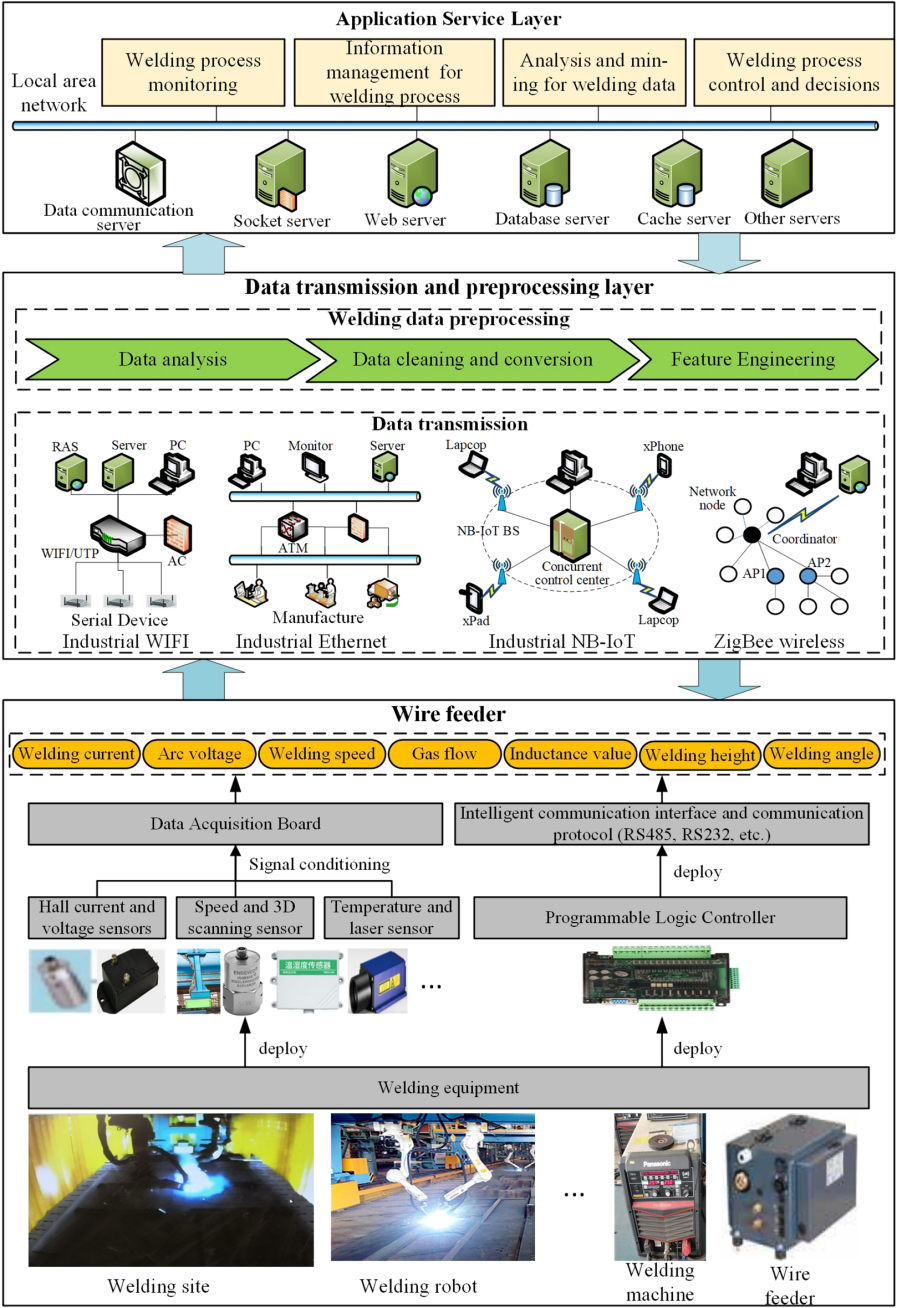
A welding process data acquisition and processing framework.

The collection and transmission of process parameters during the welding process is an important basis for supporting the real-time prediction of welding quality. Therefore, a welding data acquisition and processing framework for ship component based on the IoT system is proposed, which is mainly divided into three levels: data perception, data transmission and preprocessing, and application services, as shown in Fig. 2.

During the execution of the welding process, the data sensing layer is mainly responsible for collecting various multi-source heterogeneous data in real-time and accurately, and providing a stable original data source for the data integration and analysis phase. The sensing data types mainly include welding process parameters, operating status information of welding equipment, and welding quality indicators. The collection method can be used through interface and protocol conversion or by connecting to an external intelligent sensing device. For example, for some non-digital smart devices, data collection can be operated by analog signals of electrical circuits. Then, data such as current, voltage, and welding speed are collected from the welding equipment by installing sensors such as current, voltage, and speed. Finally, a data acquisition board, such as PCL-818L, is used for analog-to-digital conversion, summary fusion, and data transmission. For most digital intelligent devices, data collection can use various communication interfaces or serial ports, PLC networks or communication interfaces, and other methods to collect and summarize the execution parameters and operating status of the equipment. Then, through the corresponding communication protocol, such as OPC-UA, MQTT, etc., the data read and write operations among the application, the server, and the PLC are realized.

The data transmission layer is mainly responsible for transmitting multi-source heterogeneous welding data collected on-site, achieving interconnectivity between underlying devices, application services, and multiple databases. As the new generation of communication technology matures, there are many ways to choose from for industrial-level information communication, such as 5G, Zigbee, industrial WiFi networks, and industrial Ethernet. According to actual needs and complementary advantages, a combined communication scheme can also be formed to meet the requirements of transmission and anti-interference ability, communication speed, and stability. The application scene and system functional requirements of this study are taken into account. Choose the combination of wired communication technology and wireless communication technology applications. To achieve efficient deployment of communication networks, with real-time welding data fast and stable transmission and portable networking.

The diversity of equipment in shipbuilding workshops and the heterogeneity of application systems have caused data to have multi-source and heterogeneous characteristics. Therefore, data integration is to shield the differences in data types and structures to realize unified storage, management, and data analysis. The key technologies of data integration include data storage and management, as well as data preprocessing. Among them, data storage management is the basis for maximizing data value and data preprocessing. Standard database technologies include SQL databases, such as MySQL, Oracle, etc., Redis, HBase, and other types of NoSQL databases. The specific deployment can be mixed and used according to actual needs and application scenarios to achieve complementary advantages and maximize benefits.

### Data feature selection

Data feature selection is the premise to ensure the quality of data analysis and mining. It can not only ensure the quality and uniform format of the perceived data set, but also effectively avoid the feature jumble and curse of dimensionality in the process of data analysis. The welding data collected on-site will inevitably have characteristics such as missing, non-standard, and large capacity, requiring data filtering, data recovery, and data conversion to improve data quality and unify data formats.

The Relief-F algorithm is obtained by extending the function of the Relief algorithm by I. Kononenko^44^. It is a feature weight algorithm. Its function is to assign different weights to feature quantities based on the correlation between each feature quantity and category. Remove feature quantities with weights less than a certain threshold based on the calculation results. To achieve optimization of feature quantities. For multi-classification problems, suppose that the single-label training data $D=\{\left({x}_{1},{y}_{1}\right),\left({x}_{2},{y}_{2}\right),\dots ,({x}_{n},{y}_{n})\}$ set can be divided into $|c|$ categories. Relief-F can find the nearest neighbor examples in the sample set of class ${K}_{j}({K}_{j}\in ,\{\mathrm{1,2},\dots ,|c|\})$ and each other class for the example ${X}_{i}$ belonging to class ${K}_{j}$. Suppose that the near-hit examples of ${X}_{i}$ is ${X}_{i,l,nh}$ ($l=\mathrm{1,2},\dots ,\left|c\right|;l\ne {K}_{j}$) and the near-miss examples of ${X}_{i}$ is ${X}_{i,l,nm}$. Then, the iterative calculation formula is used to update the feature weight $W(A)$ of the attribute feature A. According to the input data set $D$, set the sampling times of the sample to $m$, the threshold of the feature weight to $\delta$, and the number of nearest neighbor samples to $k$, and the corresponding calculation description is as follows:

1. The feature weight $W(A)$ of each attribute is initialized to 0, and the feature weight set $T$ of the sample data set $D$ is an empty set.
2. Starting iterative calculation, and randomly selecting example ${X}_{i}$ from the sample data set $D$.
3. From the sample set $D$ of the same type as ${X}_{i}$, finding $k$ the near-hit examples ${X}_{i,l,nh}$, denoted as ${H}_{i}(c)(i=\mathrm{1,2},\dots ,k,c=class({X}_{i}))$. From the sample set $D$ of the same different type as ${X}_{i}$, finding $k$ the near-miss examples ${X}_{i,l,nm}$, denoted as ${M}_{i}(\widehat{c})(\widehat{c}\ne class({X}_{i}))$.
4. Updating the feature weights $W(A)$ and $T$, the calculation formulas are as follows:
  1 $$ W\left( A \right) = W\left( A \right) - \frac{{\mathop \sum \nolimits_{i = 1}^{k} diff\left( {A,X_{i} ,H_{i} \left( c \right)} \right)}}{m \times k} + \frac{{\mathop \sum \nolimits_{{\hat{c} \ne class\left( {X_{i} } \right)}} \left[ {\frac{{P\left( {\hat{c}} \right)}}{{1 - P\left( {class\left( {X_{i} } \right)} \right)}}\mathop \sum \nolimits_{i = 1}^{k} diff\left( {A,X_{i} ,M_{i} \left( {\hat{c}} \right)} \right)} \right]}}{m \times k} $$
  2 $$ diff\left( {A,X_{1} ,X_{2} } \right) = \left\{ {\begin{array}{*{20}c} {\frac{{\left| {X_{1} \left[ A \right] - X_{2} \left[ A \right]} \right|}}{\max \left( A \right) - \min \left( A \right)},\;If\;feature\;A\;is\;continuous} \\ {0,\;If\;feature\;A\;is\;discrete,\;and\;X_{1} \left[ A \right] = X_{2} \left[ A \right]\# } \\ {1,\;If\;feature\;A\;is\;discrete,\;and\;X_{1} \left[ A \right] \ne X_{2} \left[ A \right]} \\ \end{array} } \right. $$

where $diff\left(A,{X}_{1},{X}_{2}\right)$ represents the distance between the sample ${X}_{1}$ and ${X}_{2}$ on the feature $A$. $class\left({X}_{i}\right)$ represents the class label contained in sample ${X}_{i}$.$P\left(c\right)$ represents the prior probability of the result label c.

According to the weight calculation results of each attribute, the feature set of the initial input data is filtered reasonably. Specifically, a threshold $\tau$ needs to be specified, and the setting principle of its value should conform to Chebyshev's inequality $0<\tau \ll 1/\sqrt{\alpha m}$, $a$ is the probability of accepting irrelevant features and $m$ is the number of welding data samples.

## Construction the welding quality prediction model

### The welding quality prediction model based on APB

The BPNN is the most successful learning algorithm for training multi-layer feedforward neural networks. Mathematically express the principle of iterative computation of BP neural network^45^. Assume that the sample dataset $D=\left\{\left({x}_{1}{,y}_{1}\right),\left({x}_{2}{,y}_{2}\right),\dots ,\left({x}_{n}{,y}_{n}\right)\right\},{x}_{i}\in {R}^{m},{y}_{i}\in {R}^{z})$, where the input sample vector includes *m* feature attributes and outputs a *z*-dimensional real-valued vector. *m* input neural nodes, *q* hidden layer neural nodes and *z* output neural nodes form a classical error BPNN structure. Taking the three-layer multilayer feedforward network structure as an example. The threshold value of the *h*-th neural node in the hidden layer is ${\gamma }_{h}$. Threshold value of the *j-*th neural node in the output layer be ${\theta }_{j}$. Connection weights between the *i*-th neural node in the input layer and the *h*-th neural node in the hidden layer are denoted as ${v}_{ih}$. Connection weights between the *h-*th neural node in the hidden layer and the *j*-th neural node in the output layer are denoted as ${\omega }_{hj}$. Notate that *k* is the number of training iterations of the network model.

The input vectors of each neural node in the hidden layer can be computed through the threshold ${\gamma }_{h}$ and the connection weights ${v}_{ih}$ between the input layer and the hidden layer ${O}_{h}$. d is then used to generate the output vectors f of each neural node in the hidden layer by calculating through the activation function e.

The input vector ${O}_{h}$ of each neural node in the hidden layer can be calculated by the threshold ${\gamma }_{h}$ and the connection weight ${v}_{ih}$ between the input layer and the hidden layer. The output vectors ${S}_{h}$ of each neural node in the hidden layer are then computed by using ${O}_{h}$ through the activation function $L(x)$ to generate the output vectors f of each neural node in the hidden layer:

3 $$O_{h} \left( k \right) = \mathop \sum \limits_{i = 1}^{m} v_{ih} \left( k \right)D - \gamma_{h} \left( k \right),h = 1,2, \ldots q$$

4 $$S_{h} \left( k \right) = L\left( {O_{n} \left( k \right)} \right),h = 1,2, \ldots q$$

Then, by utilizing the output vectors of the implicit layer, the connection weights ${\omega }_{hj}$ and the threshold ${\theta }_{j}$, the input vector ${\beta }_{j}$ of each neural node in the output layer can be calculated. In employing the input vectors ${\beta }_{j}$ by means of the activation function $p(x)$ the output response vectors ${T}_{j}$ of each neural node in the output layer can be computed:

5 $$\beta_{j} \left( k \right) = \mathop \sum \limits_{i = 1}^{q} \omega_{hj} \left( k \right)S_{i} \left( k \right) - \theta_{i} \left( k \right),j = 1,2, \ldots z$$

6 $$T_{j} \left( k \right) = p\left( {\beta_{j} \left( k \right)} \right),j = 1,2, \ldots z$$

For training sample $\left({x}_{i}{,y}_{i}\right)$, the output vector of the error back-propagation neural network is assumed to be ${T}_{j}$. That is, the mean square error ${E}_{i}$ between the actual output value ${T}_{i}$ and the expected output value ${y}_{i}$ of the input training sample $\left({x}_{i}{,y}_{i}\right)$ can be calculated as:

7 $$E_{i} = \frac{1}{2}\mathop \sum \limits_{i = 1}^{z} (T_{i} - y_{i} )^{2}$$

The BP neural network is an iterative learning algorithm. Based on the gradient descent strategy in each round of iteration for any parameter $\delta$ the update formula is:

8 $$\delta \leftarrow \delta + \Delta \delta$$

The learning rate is given as $\eta$, and the formula is derived in terms of the incremental weight $\Delta {V}_{ih}$ of the connection between the input and hidden layers. Consider that ${V}_{ih}$ successively affects the input and output vectors of the *h*-th neural node of the hidden layer. Then it affects the input and output vectors of the *j*-th neural node of the output layer. Finally, it affects ${E}_{i}$. That is:

9 $$\frac{{\partial E_{i} }}{{\partial V_{ih} }} = \mathop \sum \limits_{j = 1}^{z} \frac{{\partial E_{i} }}{{\partial T_{j} }}\frac{{\partial T_{j} }}{{\partial \beta_{j} }}\frac{{\partial \beta_{j} }}{{\partial S_{h} }}\frac{{\partial S_{h} }}{{\partial O_{h} }}\frac{{\partial O_{h} }}{{\partial V_{ih} }} = \mathop \sum \limits_{j = 1}^{z} \frac{{\partial E_{i} }}{{\partial T_{j} }}\frac{{\partial T_{j} }}{{\partial \beta_{j} }}\frac{{\partial \beta_{j} }}{{\partial S_{h} }}\frac{{\partial S_{h} }}{{\partial O_{h} }}x_{i}$$

It is assumed that a typical Sigmoid function is used for both hidden and output layer neural element nodes. That is, there is a characteristic function formula relationship. That is:

10 $$f^{\prime}\left( x \right) = f\left( x \right)\left( {a - f\left( x \right)} \right)$$

11 $$g_{i} = - \frac{{\partial E_{i} }}{{\partial T_{j} }}\frac{{\partial T_{j} }}{{\partial \beta_{j} }} = - \left( {T_{j} - y_{j} } \right)f^{\prime}\left( {\beta_{j} - \theta_{j} } \right) = T_{j} \left( {1 - T_{j} } \right)\left( {y_{j} - T_{j} } \right)$$

Substituting into Eq. (9), the update equation for a can be solved. Similarly, updated formulas for $\Delta {\omega }_{hj}$, $\Delta {\theta }_{j}$, and $\Delta {\gamma }_{h}$ can be obtained. That is:

12 $$\Delta V_{ih} = \eta x_{i} S_{h} \left( {1 - S_{h} } \right)\mathop \sum \limits_{j = 1}^{z} \omega_{hj} g_{j}$$

13 $$\Delta \omega_{hj} = \eta g_{i} S_{h}$$

14 $$\Delta \theta_{j} = - \eta g_{i}$$

15 $$\Delta \gamma_{h} = - \eta S_{h} \left( {1 - S_{h} } \right)\mathop \sum \limits_{j = 1}^{z} \omega_{hj} g_{j}$$

**Figure 3:**
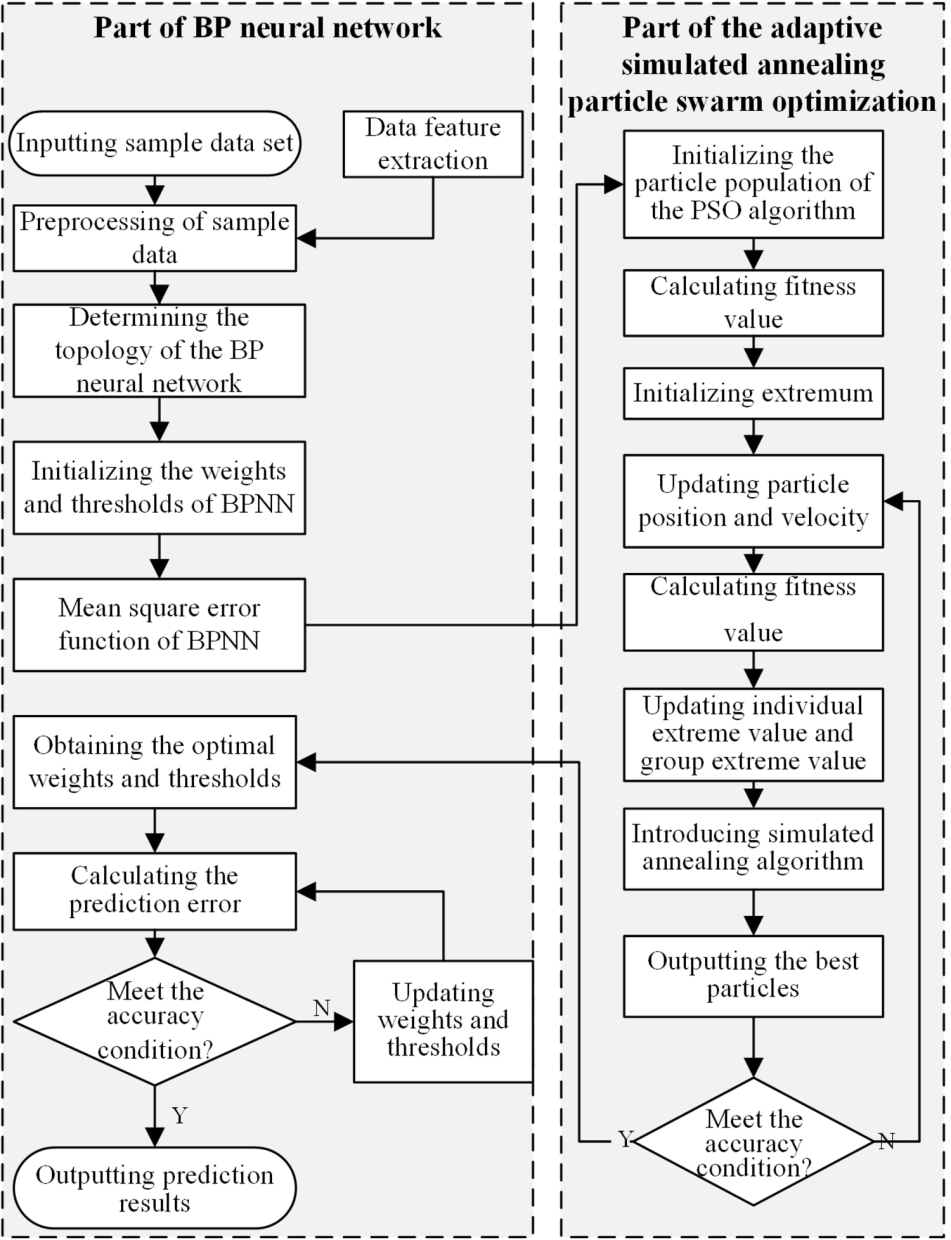
The APB algorithm flow.

The BPNN model can realize any complex mapping of multidimensional and nonlinear functions, but it is easy to fall into the optimal local solution. The particle swarm optimization is a global random search algorithm based on swarm intelligence. It has good global search performance and universality for solving the global optimal solution of multiple objective functions and constraints. It can improve the convergence accuracy of BPNN and improve prediction performance. Therefore, fusing an adaptive simulated annealing, the particle swarm optimization and the back propagation neural network, a welding quality prediction algorithm is created. The algorithm flow is shown in Fig. 3.

During the iteration optimization of the algorithm, the particle updates its position by tracking the individual extremes of the particle itself and the global extremes of the population. The movement of particles is composed of three parts, which reflect the trend of maintaining the previous speed, approaching the best position in history, group cooperation, and information sharing. The updated formulas of particle velocity and function are as follows:

16 $$v_{i} \left( {k + 1} \right) = wv_{i} \left( k \right) + c_{1} r_{1} \left( {P_{best.i} \left( k \right) - x_{i} \left( k \right)} \right) + c_{2} r_{2} \left( {G_{best} \left( k \right) - x_{i} \left( k \right)} \right)$$

17 $$x_{i} \left( {k + 1} \right) = x_{i} \left( k \right) + v_{i} \left( {k + 1} \right)$$

where the critical parameters of each part are: $w$ is the inertia weight coefficient. ${c}_{1}$ and ${c}_{2}$ are self-cognitive factors and social cognitive factors, respectively. ${v}_{i}(k)$ and ${x}_{i}\left(k\right)$, respectively represent the velocity and position of the particle $i$ at the k-th iteration. ${r}_{1}$, ${r}_{2}$ are uniform random numbers in the range of $[\mathrm{0,1}]$.${P}_{best.i}\left(k\right)$ and ${G}_{best}(k)$ represent the individual optimal solution and the optimal global solution of the particle $i$ at the k-th iteration.

$w$, ${c}_{1}$ and ${c}_{2}$ are essential parameters for controlling the iteration of the particle swarm optimization algorithm (PSO). $w$ contains the inertia of the particle flight and the strength of the algorithm's searchability. ${c}_{1}$, ${c}_{2}$ directly affect the particle's motion bias toward individual or group optimal. Therefore, to realize the adaptability of PSO, this study dynamically adjusts $w$ and ${c}_{1}$, ${c}_{2}$ to control the local and global optimization search strategy and collaborative sharing ability of the algorithm during iterative calculation. The nonlinear control strategy of a negative double tangent curve is adopted to control the change of $w$. The values of ${c}_{1}$ and ${c}_{2}$ vary with the iterative times $k$ of PSO. The updated formulas of related parameters are as follows:

18 $$\begin{array}{*{20}c} {w = \frac{{w_{max} + w_{min} }}{2} + \frac{{\tanh \left( { - 4 + 8 \times \frac{{k_{max} - k}}{{k_{max} }}} \right)\left( {w_{max} - w_{min} } \right)}}{2}} \\ \end{array}$$

19 $$\begin{array}{*{20}c} {c_{1} = c_{1max} - \frac{{k\left( {c_{1max} - c_{1min} } \right)}}{{k_{max} }}} \\ \end{array}$$

20 $$\begin{array}{*{20}c} {c_{2} = c_{2min} - \frac{{k\left( {c_{2min} - c_{2max} } \right)}}{{k_{max} }}} \\ \end{array}$$

where ${w}_{max}$ and ${w}_{min}$ are the maximum and minimum values of the inertia weight coefficient. $k$ is the current number of iterations.${k}_{max}$ is the maximum number of iterations. ${c}_{1max}$, ${c}_{1min}$ are the maximum and minimum values of the self-cognitive factor. ${c}_{2max}$, ${c}_{2min}$ are the maximum and minimum values of the social cognitive factor.

In addition, to improve the search dispersion of the PSO algorithm and avoid convergence to local minima, SA is applied to the cyclic solution process of the PSO algorithm. The SA algorithm is an adaptive iterative heuristic probabilistic search algorithm. It has strong robustness, global convergence, computational parallelism, and adaptability, and can be suitable for solving nonlinear problems, as well as solving different types of design variable optimization problems. The specific process of the algorithm is as follows:

1. Select welding quality influencing factors with strong correlation as input characteristic set and corresponding welding quality data as output attribute set to establish training data set and verification data set of algorithm;
2. Preliminary construction of the BPNN prediction model for welding quality prediction;
3. The suitability function is set as the mean square error calculation function to evaluate the predictive performance. The flying particles are the weights and threshold parameter matrices of each neural network node. Particle population size N and maximum evolution number M are initialized. Set the search space dimension and speed range of the particle swarm. Random updating of the positions and velocities of all particles in the population;
4. Calculate the fitness values of all initial particles in the population, compare the optimal position of particle individual ${{\text{P}}}_{best}$ with the optimal position of population ${{\text{G}}}_{best}$, and set the initial temperature of simulated annealing algorithm $T\left(0\right)$ according to formula (22);
5. Update the position and velocity of the particles by adjusting w, ${c}_{1}$, and ${c}_{2}$ adaptively according to formulas (18), (19), and (20). Perform an iterative optimization. Update the global optimum of the population;
6. Set $T=T(0)$ and initial solution ${S}_{1}$ as the global optimal solution, and determine the number of iterations at each temperature T, denoted as the chain length L of the Metropolis algorithm;
7. A stochastic perturbation is added to solution ${S}_{1}$ of the wheeled iteration and a new solution ${S}_{2}$ is generated.
8. Calculate the increment $df=f{(S}_{2}$)$-f({S}_{1})$ of the new solution ${S}_{2}$, where $f(x)$ is the fitness function;
9. If $df<0$, ${S}_{2}$ is accepted as the current solution for the iteration of the current wheel, so ${{S}_{1}=S}_{2}$. If $df>0$, then the acceptance probability ${\text{exp}}(df/T)$ of ${S}_{2}$ is calculated, i.e. the random number Rand with uniform distribution is randomly generated in the interval (0,1). When the acceptance probability ${\text{exp}}(df/T)>rand$, ${S}_{2}$ is also accepted as the new solution for the iteration, otherwise the current solution ${S}_{1}$ is retained;
10. The predictive error of the current solution ${S}_{1}$ has reached the accuracy requirement, or the number of iterations of the algorithm reaches the maximum number of iterations M, and the algorithm terminates. Otherwise, the algorithm decays the current temperature T according to the set attenuation function and returns to step 5 for cycle iteration until the condition is met and the current global optimal solution is output;
11. Output the current optimal particle, i.e. the optimal threshold and weight vector, fit the validation sample set and calculate the forecast error, and return to step 5 if conditions are not met.

After each iteration, the algorithm simulates the linear decay process of the initial temperature $T\left(0\right).$ Then, the algorithm can not only accept the optimal solution, but also accept a certain probability ${P}_{T}$, which improves the ability of PSO to jump out of the optimal local solution in the iterative optimization process. The updated formulas of related parameters are as follows:

21 $$p_{T\left( k \right)} \left( i \right) = \left\{ {\begin{array}{*{20}l} {1,} \hfill & {f\left( {X_{i + 1}^{T\left( k \right)} } \right) \ll f\left( {X_{i}^{T\left( k \right)} } \right)} \hfill \\ {exp\left( {f\left( {X_{i + 1}^{T\left( k \right)} } \right) - \frac{{f\left( {X_{i}^{T\left( k \right)} } \right)}}{T\left( k \right)}} \right),} \hfill & {f\left( {X_{i + 1}^{T\left( k \right)} } \right) > f\left( {X_{i}^{T\left( k \right)} } \right)} \hfill \\ \end{array} } \right.$$

22 $$T\left( k \right) = \left\{ {\begin{array}{*{20}c} {G_{best} /log\left( {0.2} \right), k = 1} \\ {T\left( {k - 1} \right)*\mu , k > 1} \\ \end{array} } \right.$$

where ${X}_{i+1}^{T(k)}$ represents the individual solution at the current temperature $T\left(k\right)$. ${P}_{T\left(k\right)}(i)$ is an acceptable probability that the new solution ${X}_{i+1}^{T(k)}$ can replace the historical solution ${X}_{i}^{T(k)}$. $T\left(k\right)$ represents the current temperature of the k-th annealing. $\mu$ represents the cooling coefficient.

To evaluate the prediction accuracy of the improved algorithm model, the coefficient of determination (R^2^), the mean absolute percentage error (MAPE), and the root mean square error (RMSE) is selected as a predictor of error in this thesis. The specific reference formula is:

10 $$R^{2} = 1 - \frac{{\mathop \sum \nolimits_{i = 1}^{n} \left( {y_{i} - \hat{y}_{i} } \right)^{2} }}{{\mathop \sum \nolimits_{i = 1}^{n} \left( {y_{i} - \overline{y}} \right)^{2} }}$$

11 $$\begin{array}{*{20}c} {MAPE = \frac{1}{m}\mathop \sum \limits_{i = 1}^{m} \left| {\frac{{y^{\left( i \right)} - h\left( {X^{\left( i \right)} } \right)}}{{y^{\left( i \right)} }}} \right| \times 100\% } \\ \end{array}$$

12 $$\begin{array}{*{20}c} {RMSE = \sqrt {\frac{1}{m}\mathop \sum \limits_{i = 1}^{m} \left( {h\left( {X^{\left( i \right)} } \right) - y^{\left( i \right)} } \right)^{2} } } \\ \end{array}$$

where n is the sample size in the dataset; ${y}_{i}$ is the actual observation corresponding to the ith sample instance; ${\widehat{y}}_{i}$ is the fitted prediction corresponding to the ith sample instance; and $\overline{y }$ is the average observation of the n sample instances. ${y}^{(i)}$ is the actual value corresponding to the i-th instances. $h({X}^{(i)})$ is the predicted value corresponding to the i-th instances.

The R^2^ indicates the superiority of fitting the covariance between the sample independent variables and the dependent variable in the regression model. The MAPE and RMSE reflect the degree of deviation between the predicted and actual values.

### Process parameter optimization

The genetic algorithm is first proposed by John Holland^46^ according to the evolution law of biological populations in nature. It is an algorithm to obtain the optimal solution by simulating the natural evolution of the biological population. It can handle complex nonlinear combinatorial optimization problems and has a good global optimization-seeking ability, so genetic algorithm is widely used in optimization problems in many engineering fields. Based on the welding quality prediction model built in the previous chapter, the genetic algorithm is introduced to optimize the welding process parameters to obtain the optimal combination of process parameters.

**Figure 4:**
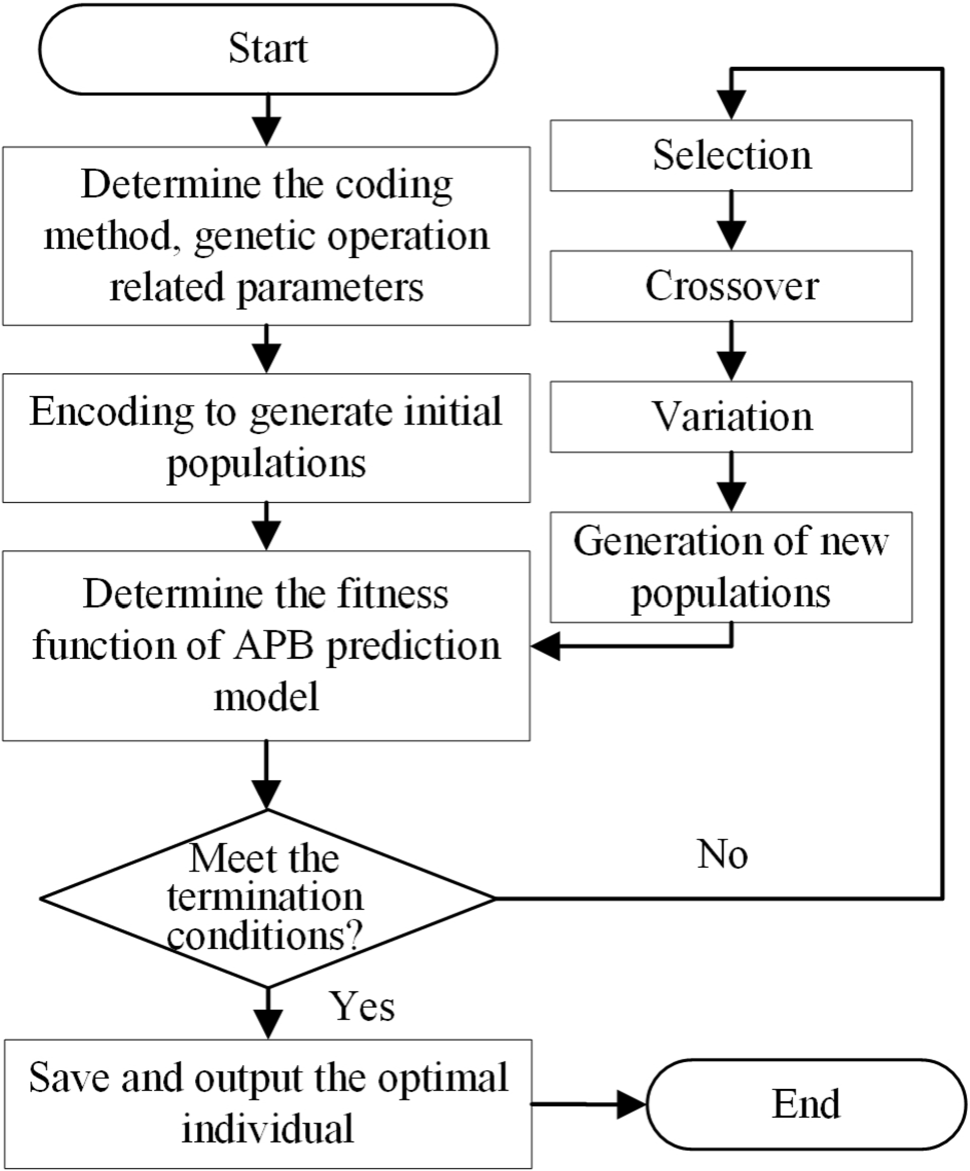
The optimization process of welding process parameters.

The specific idea is the welding current, arc voltage, welding speed, wire elongation, welding gas flow, and inductance of each process parameter by the actual number encoding as a gene composition chromosome. A chromosome represents a set of welding process parameters combined, to obtain the initial population, and then through selection, crossover, mutation to generate new populations, and according to the above-established prediction model for the degree of adaptation function for evaluation, and then finally iterate through the genetic algorithm to get the optimal combination of process parameters. The specific algorithm flow is shown in Fig. 4.

## Case study

To demonstrate the feasibility of the method proposed in this paper, welding experiments on ship unit components are conducted in cooperation with a large shipyard. The proposed method is verified to accurately predict the welding quality for ship unit-assembly welding.

### Obtain the welding process data

**Figure 5:**
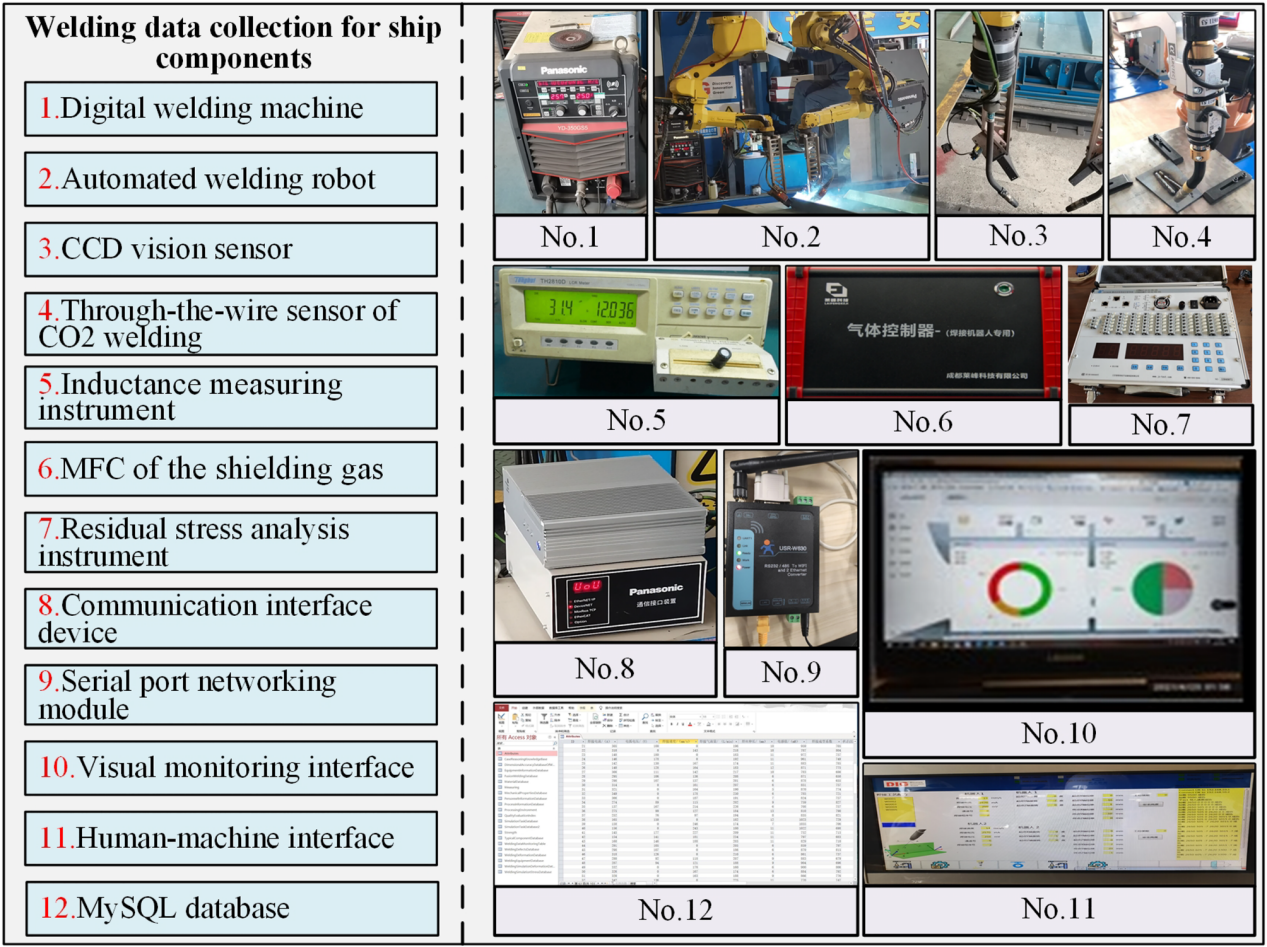
The welding data acquisition system of the ship component structure.

Based on the industrial IoT framework of data acquisition and processing of ship component welding, The welding data collection method is validated, as shown in Fig. 5 (The clearer version is shown in Supplementary Figure S2). The ship plate used in the investigation is general strength hull structural steel-Q235B. Its specific size is 300 mm × 150 mm × 5 mm, and its welding process chooses the center surfacing welding of the ship component. In the welding experiment of the ship component structure, the digital welding machine selected is Panasonic's all-digital Metal Inert Gas welding machine, model YD-350GS5 of the GP5 series, which has a built-in IoT module and analog communication interface. The automatic welding robot uses a Panasonic TAWERS welding robot, which can realize very low spatter welding of ship components. To collect key welding process parameters, some intelligent sensors and precision measuring instruments are equipped in the experiment. The threading sensor of CO2 welding can monitor the elongation of welding wire during welding. TH2810 inductance measuring instrument is used to measure the inductance during welding. In addition, the mass flow controller of shielding gas is used to measure the welding gas flow in the welding process (More complete description is shown in Supplementary Table S1).

The equipment used for welding data transmission includes communication interface equipment and a serial port network module. For digital welding machines and welding robots, PLC provides analog input modules that can receive standard voltage or current signals converted by transmitters. Then, after the calculation and analysis of PLC, the analyzed data can be displayed on the human–machine interface (HMI) of the welding site through the communication interface device and communication protocol. The intelligent sensor configured in the experiment has its communication interface, such as RS232 and RS485. Therefore, the serial port network module can establish a connection and data protocol conversion with each sensor. Wireless Fidelity transmission and Ethernet can be set through a radiofrequency (RF) antenna and WAN/LAN conversion component to support the welding data reading and writing operation between welding site and the upper computer. In this case, MySQL database is used to store, manage and share welding data.

Residual stresses are measured by the blind hole method on the finished welded steel plate. The value of the residual stress reflects the quality of the weld. The blind hole method is based on applying a strain gauge to the surface of the workpiece to be measured. Then a hole is punched into the workpiece to cause stress relaxation around the hole and generate a new stress field distribution. Strain release is collected by the strain gauge, and the original residual stress and strain of the workpiece can be deduced based on the principle of elasticity.

**Figure 6:**
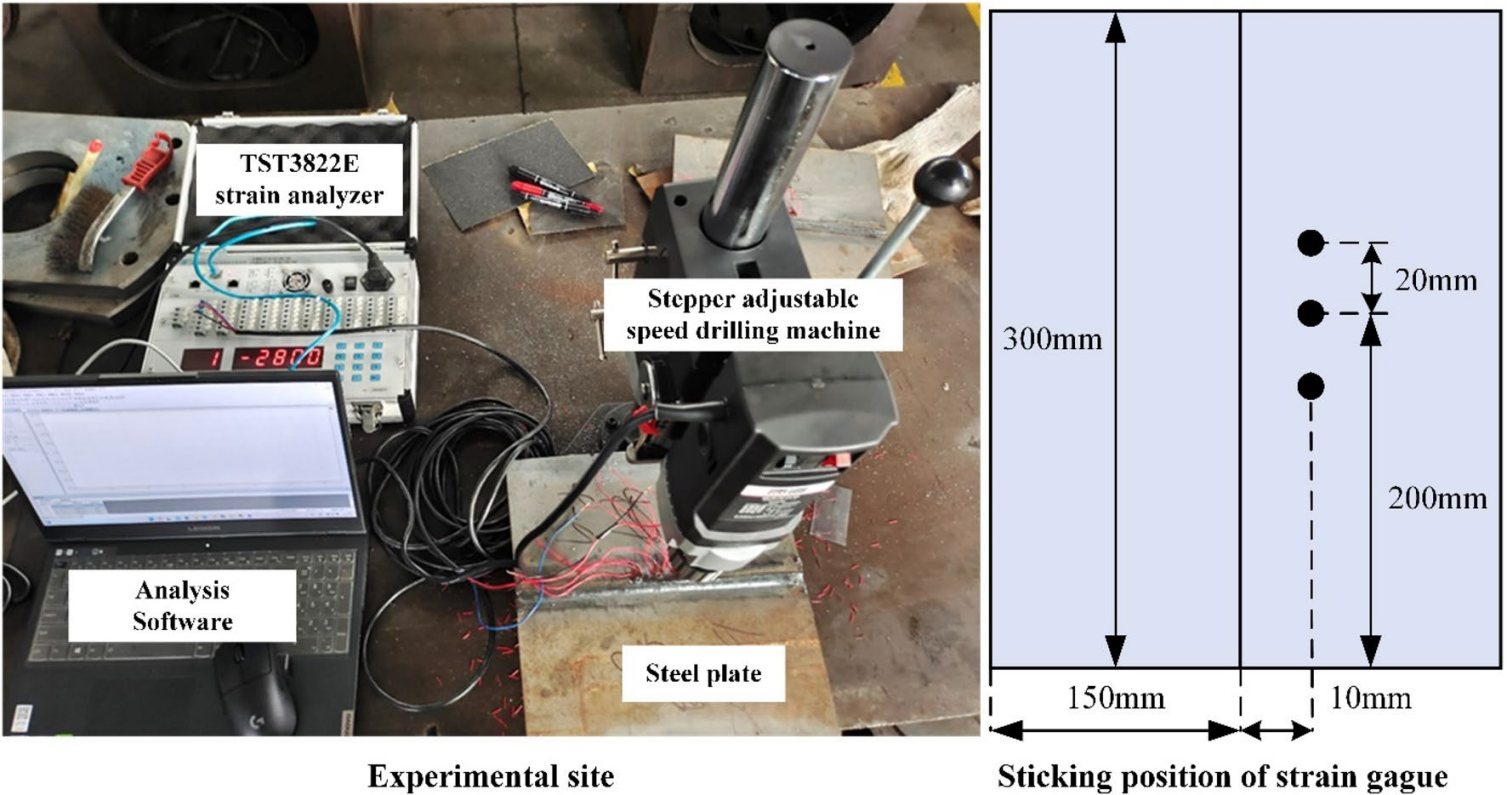
Blind hole method to collect residual stress.

The measurement equipment consisted of a stepper-adjustable drilling machine, a three-phase strain gauge, a TST3822E static strain test analyzer, and software. The diameter of the blind hole is 2 mm, and the depth of the hole is 3 mm. The measured stress is the average value of the pressure distribution in the depth of the blind spot. According to the direction of action, the residual welding stresses are divided into longitudinal residual stresses parallel to the weld axis and transverse residual stresses perpendicular to the weld. In this experiment, the strain gauge type is chosen as a three-phase right-angle strain gauge. That is, the layout angles of the strain gauges are 0°, 45°, and 90°. Longitudinal strain, principal strain, and transverse strain are measured, respectively. Since the distribution of longitudinal residual stresses is more regular than that of transverse residual stresses, only the themes in the 0° direction are considered in this experiment. The amount of strain changes along the weld direction, and then the analysis software yields the longitudinal residual stress. As shown in Fig. 6, the operation site and the sticking position of the strain gauge for the experiment using the blind hole method are used. The participating stresses of each plate are collected through the TST3822E static strain test analyzer and computer software.

### Preprocessing the welding process data

**Figure 7:**
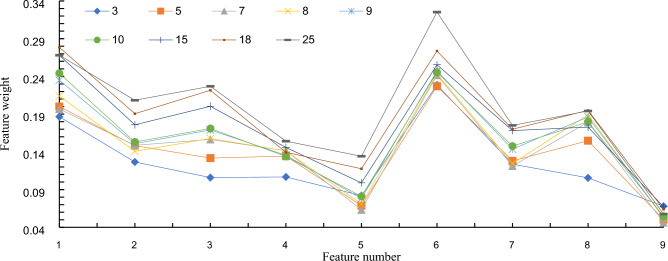
The final weight of each data feature.

According to the correlation between the collected welding data and weld formation quality, MATLAB software and the Relief-F algorithm assign different influence weights to each data feature. Data features whose weight is less than the threshold value, such as data types irrelevant or weakly related to the weld formation quality, these data features will be excluded. The collected data includes welding current, arc voltage, welding speed, welding gun angle, steel plate thickness, welding gas flow, welding wire diameter, inductance value, and welding wire elongation. The Relief-F algorithm needs to set the number of neighbors and sampling times. Combined with the experimental sample data collected in the experiment, this case selects the number of neighbors $k=\mathrm{3,5},\mathrm{7,8},9,\mathrm{10,15,18,25}$, and the number of sampling $m$ is 80. The calculation results are shown in Fig. 7. The average of the calculation results of each group is used as the final weight of each data feature, and the calculation result is shown in Table 1.

**Table 1:** The corresponding feature number and value range.

Feature number	Types of data features	Feature weight
1	Welding current/A	0.232
2	Inductance value/(H)	0.160
3	Welding gas flow/(L/min)	0.171
4	Welding gun angle/(rad)	0.136
5	Steel plate thickness/(mm)	0.088
6	Arc voltage/V	0.254
7	Welding wire diameter/(mm)	0.057
8	Welding speed/(mm/s)	0.173
9	Welding wire elongation/(mm)	0.144

Among the features of the collected welding data, the influence weights of arc voltage and welding current on the quality of weld formation are the largest, which are 0.254 and 0.232, respectively. The main reason is that when the arc voltage and welding current increase, it will directly cause the heat energy input to the weld seam and the melting amount of the welding wire to increase, thereby increasing the width, penetration, and reinforcement of the weld seam. Secondly, the data feature with a relatively small degree of influence is the welding speed, and its corresponding influence weight is 0.173. When the welding speed is too high, the cooling rate of the welding seam will be too fast. Then, it will lead to deposition and the reduction of the number of metal coatings, which will affect the quality of weld formation. On the contrary, it will cause the heat concentration and width of the molten pool to increase, resulting in burn-through and other welding defects. In addition, in CO2 gas-shielded welding, the welding gas flow rate is a key parameter that affects the quality of weld formation, and its calculated influence weight is 0.171. When the gas flow is too large, it will cause instability and disturbance of the molten pool and arc of the weld, resulting in turbulence and splashing in the molten pool. On the contrary, it will directly reduce the protective effect of gas and affect the quality of weld formation. The inductance value will affect the penetration of the weld, and its weight is calculated to be 0.16. The welding wire elongation will directly affect the protective effect of the gas, and its weight is estimated to be 0.144. The welding gun angle, steel plate thickness, and welding wire diameter also have a particular influence on the forming quality of the weld. The influence weights are calculated to be 0.13, 0.08, and 0.05, respectively.

In this verification case, the data sample size for CO2 gas-shielded welding of the ship component structure is 350, and $\alpha$ is 0.145. It is calculated that the weight threshold range of the influence weight of the weld forming quality in the CO2 gas-shielded welding of the ship component structure is $0<\tau \le 0.14$. Combined with the calculation results of the data feature weight, the influencing factors whose influence weight is greater than the threshold value are considered the main process parameters in this experiment. The main process parameters are arc voltage, welding current, welding speed, welding gas flow, inductance value, and welding wire extension length. These will be used as key input variables for constructing a welding quality prediction model.

### Predict the welding quality

Using MATLAB as the verification platform, this case uses the APB algorithm model to predict the welding quality of the ship component. 300 sets of welding data are selected to train the algorithm model (Complete data in Supplementary Table S2), and 74 sets are selected for verification. The verification data set is shown in Table 2 (Complete data in Supplementary Table S3). This case considers the weld forming coefficient as the target variable and selects six variables as the key welding quality influencing factors according to the feature selection results in “Preprocessing the welding process data” section. The six key welding quality influencing factors include welding current, arc voltage, welding speed, welding wire elongation, inductance value, and welding gas flow.

**Table 2:** The verification data set.

Sample number	Welding current/A	Arc voltage/V	Welding speed/(mm/s)	Wire elongation/(mm)	Inductance value/(H)	Welding gas flow/(L/min)	Residual stress/(Mpa)
1	161	57.8	6.1	0.122	61	14.44	222
2	149.3	60.3	4.8	0.0984	49.4	13.96	198
3	125.85	70.35	5.3	0.0448	29.3	12.62	131
4	122.35	71.85	5.21	0.368	26.3	12.42	121
5	146.85	61.35	5	0.0928	47.3	13.82	191
6	159	58.2	5.9	0.118	59	14.36	218
7	155.5	58.9	5.55	0.111	55.5	14.22	211
8	163	57.4	6.3	0.126	63	14.52	226
9	126.9	69.9	4.4	0.472	30.2	12.68	134
10	116.75	74.25	5.1	0.24	21.5	12.1	105
…	…	…	…	…	…	…	…
67	183.5	26.7	5.33	0.835	167	25.05	267
68	150	22.4	7.32	0.192	166.5	23.6	112
69	150	21.6	7.38	0.178	198	22.4	108
70	150	33	6.525	0.3775	151.2	19.8	165
71	150	23.8	7.215	0.2165	198	16.5	119
72	150	31.2	6.66	0.346	136.08	23.6	156
73	160.5	22.1	5.79	0.605	121	18	221
74	150	21.8	7.365	0.1815	153	22.7	109

After conducting many experiments using the above welding data, the upper limit ${w}_{max}$ is set to 0.9, and the lower limit ${w}_{min}$ is set to 0.4. The maximum number of ${k}_{max}$ iterations of PSO is set to 1000. The parameter combination of the self-cognitive factor and social cognitive factor is that ${c}_{1max},{c}_{1min},{c}_{2max}$, and ${c}_{2min}$ are set to 2.5, 1.25, 2.5, and 1.25, respectively. The APB algorithm's global search capability and convergence speed can be balanced and achieve better results. The Metropolis criterion of the SA algorithm is introduced into the iterative calculation of the PSO algorithm. In the case verification, the initial temperature ($T\left(0\right)={10}^{4}$) is attenuated by the cooling coefficient ($\mu =0.9$). The 24 sets of welding data in Table 2 are substituted into the trained APB algorithm model to predict and verify the forming weld coefficient. The actual output value of each verification sample is compared with the expected value, and the relative error is calculated, as shown in Table 3. (Complete data in Supplementary Table S4). In this case, the maximum and minimum relative prediction errors of the SAPSO_BPNN algorithm model on the validation sample data set are 8.764% and 5.364%, respectively. In general, the error of the proposed prediction algorithm is relatively small and can satisfy the accuracy requirements for predicting the welding quality of ship components of ships.

**Table 3:** Predicted results and relative errors of welding verification samples.

Sample number	Actual residual stress	The predicted value of the APB algorithm	Analysis error (%)
1	222	232.6586548	4.80120
2	198	184.3617309	6.88801
3	131	124.1711304	5.21288
4	121	129.667395	7.16314
5	191	180.4715942	5.51225
6	218	234.529187	7.58220
7	211	222.0158325	5.22077
8	226	207.5712036	8.15433
9	134	126.2844116	5.75790
10	105	112.8535766	7.47960
…	…	…	…
67	267	249.984624	6.37280
68	112	102.430384	8.54430
69	108	100.71648	6.74400
70	165	177.15126	7.36440
71	119	108.67794	8.67400
72	156	165.30852	5.96700
73	221	206.90462	6.37800
74	109	99.57804	8.64400

**Figure 8:**
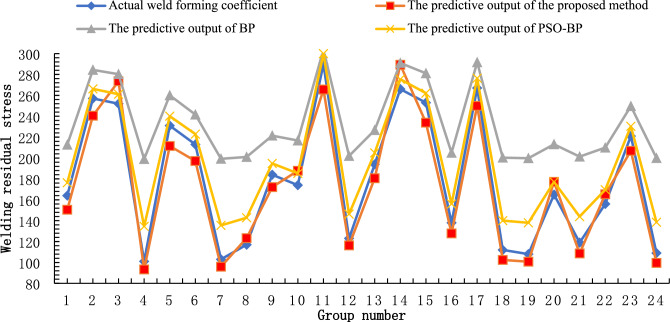
The predictive outputs and comparison result of different algorithms.

In addition, the improvements and advantages of the proposed APB algorithm model are further explained. Using the same welding data set above, the BPNN, BPNN optimized method based on the particle swarm optimization algorithm (PSO-BP), and the APB algorithm are selected to predict the residual welding stress. Some data comparison results are shown in Fig. 8.

The calculation results of the algorithm evaluation indicators R2, MAPE and RSME are shown in Table 4. In comparison, the prediction accuracy of the welding data samples using the PSO-BP algorithm is higher than that of the BPNN. In addition, the prediction accuracy based on the APB algorithm is also significantly improved compared to PSO-BP.

**Table 4:** The comparison of prediction accuracy indicators of various algorithms.

Prediction model	R^2^	MAPE (%)	RMSE
BP	0.659	39.83	0.4933
PSO-BP	0.809	12.53	0.2121
APB	0.952	1.77	0.0709

### Optimize the welding process parameters

Several workpieces with high welding residual stress are found in the experiment. The quality of these workpieces is not up to requirements, resulting in scrap. It will bring unnecessary economic loss to the enterprise. To reduce the loss and improve efficiency, the unqualified variety of welding process parameters is optimized by using the global optimization ability of the genetic algorithm.

Relevant parameters of the genetic algorithm are selected: maximum evolutionary algebra, population size, crossover probability, and variation probability are 100, 50, 0.7, and 0.01, respectively. The proposed forecast model is used as the objective function. The smaller the residual stress value, the higher the suitability. The experiment is carried out again to optimize the defective combination of process parameters in real time. The residual stress of the optimized product is re-measured. The results are shown in Table 5. The experimental results show that the optimized combination of process parameters can yield products with lower residual stress. Improve quality and reduce economic losses. It can provide a reference for the real-time improvement of the welding process in enterprises.

**Table 5:** Optimization results of welding parameters.

Sample number	Welding current/A	Arc voltage/V	Welding speed/(mm/s)	Wire elongation/(mm)	Inductance value/(H)	Welding gas flow/(L/min)	Predicted value/(Mpa)	Actual Residual stress/(Mpa)
1	150	21.2	7.34	0.162	158	21.2	97.56,634	104
2	154	20.6	7.455	0.173	162	20.9	102.71,648	107
3	150	21.8	7.365	0.165	153	21.5	95. 31,051	101

## Conclusion and future works

To meet the requirements of real-time monitoring and accurately prediction the ship unit-assembly welding quality, an IOT-based welding data acquisition framework is firstly established. And the stable and reliable data is obtained. The welding process monitoring elements are determined based on the feature dimensionality reduction methods. According to the Relief-F algorithm, the crucial features data is selected among the historical dataset. Secondly, the correlation rule between process parameters and welding quality is established. The prediction model of the ship unit-assembly welding is constructed by fusing the adaptive simulated annealing, the particle swarm optimization and the back propagation neural network. In order to optimize the welding parameters, the genetic algorithm is selected. Finally, the experimental welding of ship component is used as an example to verify the effectiveness of the proposed critical techniques.

The experimental results show that the proposed APB prediction model can predict the welding characteristics more effectively than the traditional methods, with a prediction accuracy of more than 91.236%, the coefficient of determination (R^2^) is increased from 0.659 to 0.952, the mean absolute percentage error (MAPE) is reduced from 39.83 to 1.77%, and the root mean square error (RMSE) is reduced from 0.4933 to 0.0709. Showing higher prediction accuracy. It is proved that the technique can be used for online monitoring and accurate prediction of the welding quality for ship components. It can realize real-time collection and efficient transmission of big welding data, including welding process parameters, information on the operating status of welding equipment, and welding quality indicators. In addition, with the support of new-generation information technology such as the IoT, Big data, etc. the dynamic quality data in the welding process can be tracked in real-time and fully explored to realize online monitoring and accurate prediction of welding quality. With the application and development of automated welding equipment, more welding quality data and its impact factors are obtained. With the continuous updating and mining of welding data, a more accurate prediction model of welding quality needs to be established.

To dynamic control the processing quality of the ship unit-assembly welding, the proposed method can be well carried out. However, the implementation of technology is limited by the diversity and complexity of the ship sections assembly-welding process, so more effort and innovation should be paid to solve these defects. It is necessary to improve the perception and management of real-time data in the IoT system, so as to promote the deep fusion of physical and virtual workshops, and establish a more reliable virtual model and multi-dimensional welding simulation. Meanwhile, with the support of a more complete real-time database and welding quality mapping mechanism, the ship welding quality analysis ability can be continuously enhanced, and the processing quality prediction method can be further improved and innovated.

### Supplementary Information

Supplementary Information.

## Data Availability

The datasets used and/or analysed during the current study available from the corresponding author on reasonable request.
