# Supplementary material for: Prediction and optimization method for welding quality of components in ship construction
Source: Sci Rep. 2024 Apr 23;14:9353. doi: 10.1038/s41598-024-59490-w (PMC11039705; doi:10.1038/s41598-024-59490-w)
Supplement: Supplementary file 1 — Supplementary Information. [file 41598_2024_59490_MOESM1_ESM.docx]

**Figure S1 (Picture in Figure 1)**

| 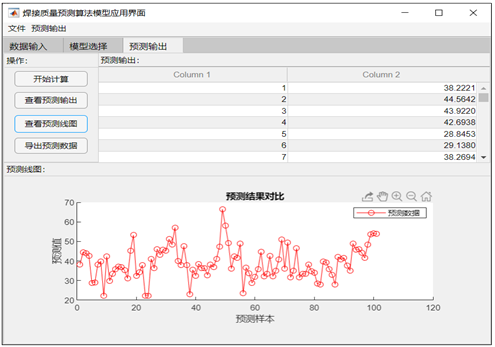**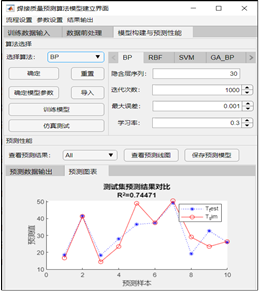** |
| --- |
| **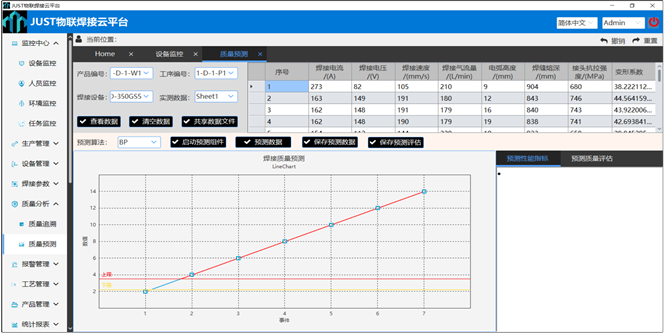** |

**Table S1 Equipment type and technical specifications**

| **Number** | **Equipment** | **Specification** | **Experiment related parameters** |
| --- | --- | --- | --- |
| 1 | Digital welding machine | Panasonic GP5 YD-350GS5 | Functions: Gas welding  Power: 13.5kW  Protective gas type:  Inert gas such as Ar, CO_2_, etc. |
| 2 | Automated welding robot | Panasonic TAWERS | Standardly equipped with MTS-CO2, SP-MAG, HD-PULSE, NORMAL-PULSE and other welding methods |
| 3 | CCD vision sensor | KEYENCE CV-020 | Measuring range: 100m  Measurement accuracy: 99%  Magnification: 500  Field of view: 50 m@100m |
| 4 | Inductance measuring instrument | Tonghui TH2810D | Accuracy: 0.1%  Communication interface: RS232C  Measuring range:  0.01uH-99999H |
| 5 | Residual stress analysis instrument | TST3822E | Measured strain range: ±20,000 με  Measurement error: Less than 0.5%±3με  Communication mode: Ethernet interface |
| 6 | Serial port networking module | USR W630 | Number of serial ports: RS232*1/RS485*1  Transmission distance:  Max. 210m  Frequency range:  2.412GHz-2.484GHz |
| 7 | Sensor | Current Sensor: TOKEN TKC400BS  Voltage sensors: TOKEN TBV200 AS12/24 | TKC400BS: Rated Current: 400A  Measuring range: 1000A  Measurement error:  ≤0.5%FS  TBV200 AS12/24: Rated input: 200V  Measuring range: 400V  Measurement error:  ≤0.2%FS |

**Figure S2 (Figure 5 in the original)**

| 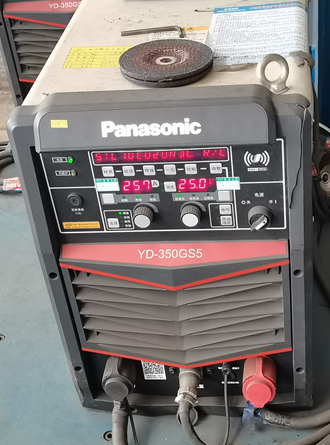  1 | 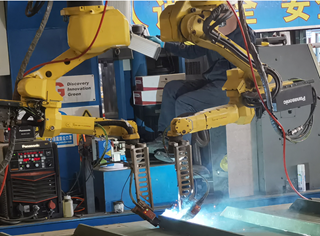  2 | | 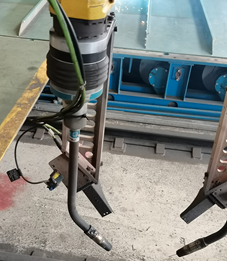  3 |
| --- | --- | --- | --- |
| 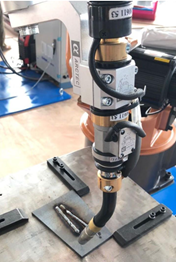  4 | 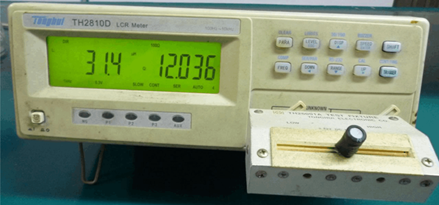  5 | | 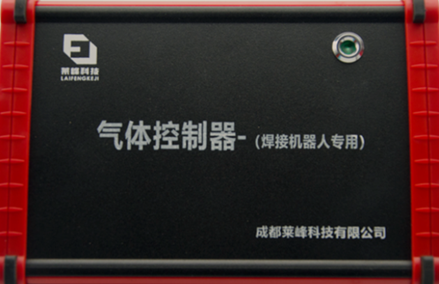  6 |
| 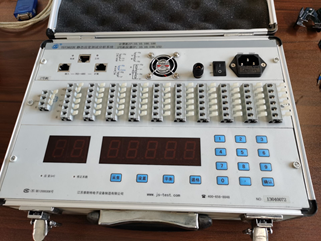  7 | 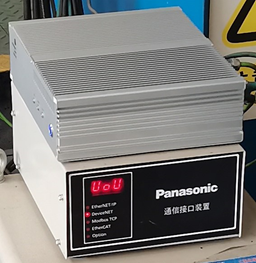  8 | | 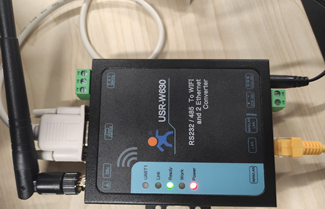  9 |
| 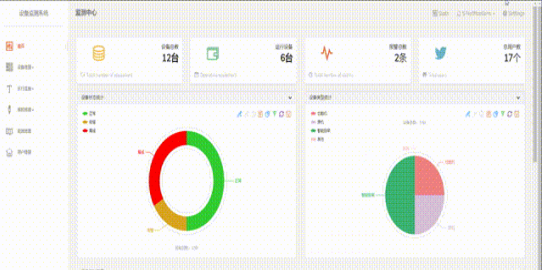  12  10 | | 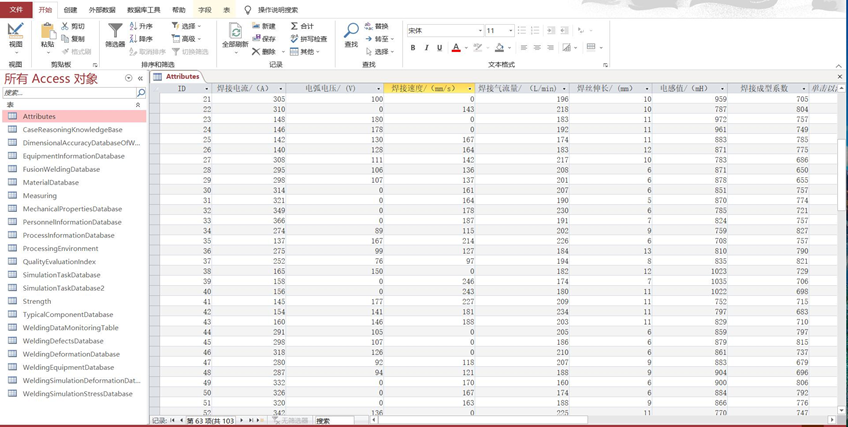  11 | |
| 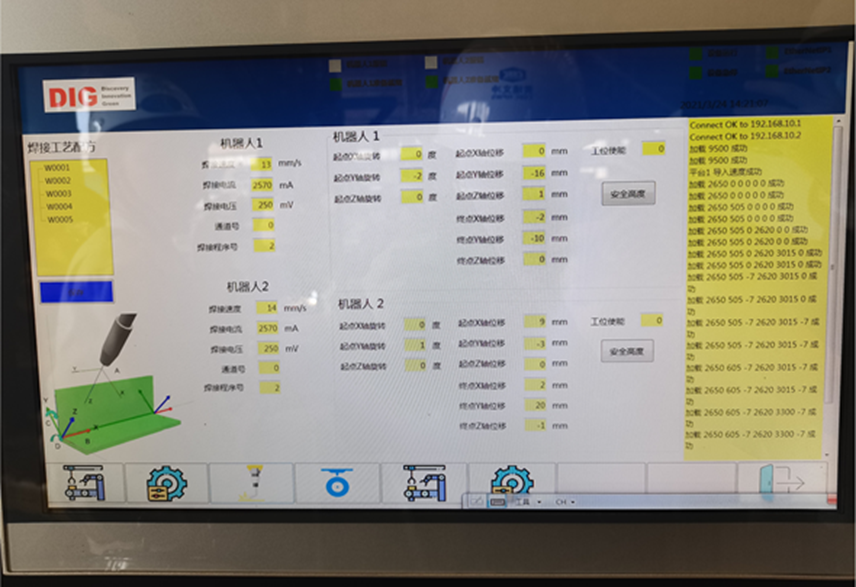 | | | |

**Table S2 Welding data set**

| Sample  number | Welding current **/A** | Arc voltage /V | Welding speed /(mm/s) | Wire elongation /(mm) | Inductance value/(H) | Welding gas flow /(L/min) | Residual stress  /(Mpa) |
| --- | --- | --- | --- | --- | --- | --- | --- |
| 1 | 161 | 57.8 | 6.1 | 0.122 | 61 | 14.44 | 222 |
| 2 | 149.3 | 60.3 | 4.8 | 0.0984 | 49.4 | 13.96 | 198 |
| 3 | 186.5 | 52.7 | 8.65 | 0.173 | 86.5 | 15.46 | 273 |
| 4 | 166 | 26.8 | 23.2 | 0.332 | 166 | 21.6 | 432 |
| 5 | 197.5 | 20.5 | 29.5 | 0.395 | 197.5 | 24.75 | 495 |
| 6 | 146.5 | 31.4 | 19.3 | 0.293 | 146.5 | 19.72 | 393 |
| 7 | 121.5 | 41.4 | 14.3 | 0.243 | 121.5 | 17.72 | 343 |
| 8 | 140 | 34 | 18 | 0.28 | 140 | 19.2 | 380 |
| 9 | 125.85 | 70.35 | 5.3 | 0.1448 | 29.3 | 12.62 | 131 |
| 10 | 180 | 24 | 26 | 0.36 | 180 | 23 | 460 |
| 11 | 128.65 | 69.15 | 5 | 0.512 | 31.7 | 12.78 | 139 |
| 12 | 122.35 | 71.85 | 5.21 | 0.368 | 26.3 | 12.42 | 121 |
| 13 | 130 | 38 | 16 | 0.26 | 130 | 18.4 | 360 |
| 14 | 151 | 29.8 | 20.2 | 0.302 | 151 | 20.1 | 402 |
| 15 | 187 | 52.6 | 8.7 | 0.174 | 87 | 15.48 | 274 |
| 16 | 126.55 | 70.05 | 5 | 0.464 | 29.9 | 12.66 | 133 |
| 17 | 175.5 | 54.9 | 7.55 | 0.151 | 75.5 | 15.02 | 251 |
| 18 | 171 | 25.8 | 24.2 | 0.342 | 171 | 22.1 | 442 |
| 19 | 146.85 | 61.35 | 5 | 0.0928 | 47.3 | 13.82 | 191 |
| 20 | 150 | 30 | 20 | 0.3 | 150 | 20 | 400 |
| 21 | 184.5 | 53.1 | 8.45 | 0.169 | 84.5 | 15.38 | 269 |
| 22 | 162 | 27.6 | 22.4 | 0.324 | 162 | 21.2 | 424 |
| 23 | 151.5 | 29.7 | 20.3 | 0.303 | 151.5 | 20.15 | 403 |
| 24 | 146.5 | 61.5 | 5 | 0.092 | 47 | 13.8 | 190 |
| 25 | 172.5 | 55.5 | 7.25 | 0.145 | 72.5 | 14.9 | 245 |
| 26 | 159 | 58.2 | 5.9 | 0.118 | 59 | 14.36 | 218 |
| 27 | 194.5 | 51.1 | 9.45 | 0.189 | 94.5 | 15.78 | 289 |
| 28 | 159 | 28.2 | 21.8 | 0.318 | 159 | 20.9 | 418 |
| 29 | 155.5 | 58.9 | 5.55 | 0.111 | 55.5 | 14.22 | 211 |
| 30 | 163 | 57.4 | 6.3 | 0.126 | 63 | 14.52 | 226 |
| 31 | 120 | 42 | 14 | 0.24 | 120 | 17.6 | 340 |
| 32 | 182 | 53.6 | 8.2 | 0.164 | 82 | 15.28 | 264 |
| 33 | 152.5 | 29.5 | 20.5 | 0.305 | 152.5 | 20.25 | 405 |
| 34 | 126.9 | 69.9 | 4.4 | 0.472 | 30.2 | 12.68 | 134 |
| 35 | 116.75 | 74.25 | 5.1 | 0.24 | 21.5 | 12.1 | 105 |
| 36 | 132 | 37.2 | 16.4 | 0.264 | 132 | 18.56 | 364 |
| 37 | 167.5 | 26.5 | 23.5 | 0.335 | 167.5 | 21.75 | 435 |
| 38 | 160 | 58 | 6 | 0.12 | 60 | 14.4 | 220 |
| 39 | 135 | 36 | 17 | 0.27 | 135 | 18.8 | 370 |
| 40 | 194.5 | 21.1 | 28.9 | 0.389 | 194.5 | 24.45 | 489 |
| 41 | 113.5 | 44.6 | 12.7 | 0.227 | 113.5 | 17.08 | 327 |
| 42 | 179.5 | 54.1 | 7.95 | 0.159 | 79.5 | 15.18 | 259 |
| 43 | 117.5 | 43 | 13.5 | 0.235 | 117.5 | 17.4 | 335 |
| 44 | 133.9 | 66.9 | 4.76 | 0.632 | 36.2 | 13.08 | 154 |
| 45 | 187.5 | 22.5 | 27.5 | 0.375 | 187.5 | 23.75 | 475 |
| 46 | 173.5 | 55.3 | 7.35 | 0.147 | 73.5 | 14.94 | 247 |
| 47 | 115.7 | 74.7 | 5 | 0.1216 | 20.6 | 12.04 | 102 |
| 48 | 178.5 | 24.3 | 25.7 | 0.357 | 178.5 | 22.85 | 457 |
| 49 | 148.25 | 60.75 | 5 | 0.096 | 48.5 | 13.9 | 195 |
| 50 | 182.5 | 53.5 | 8.25 | 0.165 | 82.5 | 15.3 | 265 |
| 51 | 159.5 | 28.1 | 21.9 | 0.319 | 159.5 | 20.95 | 419 |
| 52 | 167.5 | 56.5 | 6.75 | 0.135 | 67.5 | 14.7 | 235 |
| 53 | 134 | 36.4 | 16.8 | 0.268 | 134 | 18.72 | 368 |
| 54 | 138.45 | 64.95 | 5.1 | 0.536 | 40.1 | 13.34 | 167 |
| 55 | 120.25 | 72.75 | 4.84 | 0.032 | 24.5 | 12.3 | 115 |
| 56 | 169.5 | 56.1 | 6.95 | 0.139 | 69.5 | 14.78 | 239 |
| 57 | 167 | 56.6 | 6.7 | 0.134 | 67 | 14.68 | 234 |
| 58 | 138.8 | 64.8 | 5 | 0.744 | 40.4 | 13.36 | 168 |
| 59 | 154.5 | 29.1 | 20.9 | 0.309 | 154.5 | 20.45 | 409 |
| 60 | 157 | 58.6 | 5.7 | 0.114 | 57 | 14.28 | 214 |
| 61 | 184 | 23.2 | 26.8 | 0.368 | 184 | 23.4 | 468 |
| 62 | 118.5 | 73.5 | 5.22 | 0.028 | 23 | 12.2 | 110 |
| 63 | 199 | 20.2 | 29.8 | 0.398 | 199 | 24.9 | 498 |
| 64 | 150 | 60 | 5.14 | 0.1 | 50 | 14 | 200 |
| 65 | 133.55 | 67.05 | 4.8 | 0.624 | 35.9 | 13.06 | 153 |
| 66 | 152 | 59.6 | 5.2 | 0.104 | 52 | 14.08 | 204 |
| 67 | 167 | 26.6 | 23.4 | 0.334 | 167 | 21.7 | 434 |
| 68 | 147.5 | 31 | 19.5 | 0.295 | 147.5 | 19.8 | 395 |
| 69 | 187.5 | 52.5 | 8.75 | 0.175 | 87.5 | 15.5 | 275 |
| 70 | 108.5 | 46.6 | 11.7 | 0.217 | 108.5 | 16.68 | 317 |
| 71 | 179.5 | 24.1 | 25.9 | 0.359 | 179.5 | 22.95 | 459 |
| 72 | 125.15 | 70.65 | 5 | 0.432 | 28.7 | 12.58 | 129 |
| 73 | 160 | 28 | 22 | 0.32 | 160 | 21 | 420 |
| 74 | 178 | 24.4 | 25.6 | 0.356 | 178 | 22.8 | 456 |
| 75 | 199.5 | 20.1 | 29.9 | 0.399 | 199.5 | 24.95 | 499 |
| 76 | 166 | 56.8 | 6.6 | 0.132 | 66 | 14.64 | 232 |
| 77 | 145 | 32 | 19 | 0.29 | 145 | 19.6 | 390 |
| 78 | 192.5 | 51.5 | 9.25 | 0.185 | 92.5 | 15.7 | 285 |
| 79 | 174 | 55.2 | 7.4 | 0.148 | 74 | 14.96 | 248 |
| 80 | 111.5 | 45.4 | 12.3 | 0.223 | 111.5 | 16.92 | 323 |
| 81 | 130.4 | 68.4 | 4.92 | 0.352 | 33.2 | 12.88 | 144 |
| 82 | 191.5 | 51.7 | 9.15 | 0.183 | 91.5 | 15.66 | 283 |
| 83 | 182 | 23.6 | 26.4 | 0.364 | 182 | 23.2 | 464 |
| 84 | 186 | 22.8 | 27.2 | 0.372 | 186 | 23.6 | 472 |
| 85 | 107.5 | 47 | 11.5 | 0.215 | 107.5 | 16.6 | 315 |
| 86 | 141.6 | 63.6 | 5.7 | 0.208 | 42.8 | 13.52 | 176 |
| 87 | 139.5 | 34.2 | 17.9 | 0.279 | 139.5 | 19.16 | 379 |
| 88 | 168.5 | 26.3 | 23.7 | 0.337 | 168.5 | 21.85 | 437 |
| 89 | 105 | 48 | 11 | 0.21 | 105 | 16.4 | 310 |
| 90 | 117 | 43.2 | 13.4 | 0.234 | 117 | 17.36 | 334 |
| 91 | 135.65 | 66.15 | 5.5 | 0.372 | 37.7 | 13.18 | 159 |
| 92 | 108 | 46.8 | 11.6 | 0.216 | 108 | 16.64 | 316 |
| 93 | 188.5 | 22.3 | 27.7 | 0.377 | 188.5 | 23.85 | 477 |
| 94 | 109 | 46.4 | 11.8 | 0.218 | 109 | 16.72 | 318 |
| 95 | 156 | 58.8 | 5.6 | 0.112 | 56 | 14.24 | 212 |
| 96 | 106.5 | 47.4 | 11.3 | 0.213 | 106.5 | 16.52 | 313 |
| 97 | 126.5 | 39.4 | 15.3 | 0.253 | 126.5 | 18.12 | 353 |
| 98 | 185 | 53 | 8.5 | 0.17 | 85 | 15.4 | 270 |
| 99 | 124 | 40.4 | 14.8 | 0.248 | 124 | 17.92 | 348 |
| 100 | 173 | 55.4 | 7.3 | 0.146 | 73 | 14.92 | 246 |
| 101 | 145.5 | 31.8 | 19.1 | 0.291 | 145.5 | 19.64 | 391 |
| 102 | 174.5 | 55.1 | 7.45 | 0.149 | 74.5 | 14.98 | 249 |
| 103 | 181 | 53.8 | 8.1 | 0.162 | 81 | 15.24 | 262 |
| 104 | 140.55 | 64.05 | 4.9 | 0.484 | 41.9 | 13.46 | 173 |
| 105 | 141.95 | 63.45 | 4.65 | 0.316 | 43.1 | 13.54 | 177 |
| 106 | 190 | 22 | 28 | 0.38 | 190 | 24 | 480 |
| 107 | 139.85 | 64.35 | 6.2 | 0.468 | 41.3 | 13.42 | 171 |
| 108 | 136.35 | 65.85 | 5.7 | 0.288 | 38.3 | 13.22 | 161 |
| 109 | 139.15 | 64.65 | 5.6 | 0.352 | 40.7 | 13.38 | 169 |
| 110 | 114 | 44.4 | 12.8 | 0.228 | 114 | 17.12 | 328 |
| 111 | 164 | 57.2 | 6.4 | 0.128 | 64 | 14.56 | 228 |
| 112 | 196.5 | 20.7 | 29.3 | 0.393 | 196.5 | 24.65 | 493 |
| 113 | 132.5 | 37 | 16.5 | 0.265 | 132.5 | 18.6 | 365 |
| 114 | 115.5 | 43.8 | 13.1 | 0.231 | 115.5 | 17.24 | 331 |
| 115 | 120.5 | 41.8 | 14.1 | 0.241 | 120.5 | 17.64 | 341 |
| 116 | 153 | 59.4 | 5.3 | 0.106 | 53 | 14.12 | 206 |
| 117 | 166.5 | 56.7 | 6.65 | 0.133 | 66.5 | 14.66 | 233 |
| 118 | 191.5 | 21.7 | 28.3 | 0.383 | 191.5 | 24.15 | 483 |
| 119 | 172 | 25.6 | 24.4 | 0.344 | 172 | 22.2 | 444 |
| 120 | 132.5 | 67.5 | 5 | 0.16 | 35 | 13 | 150 |
| 121 | 126.2 | 70.2 | 4.76 | 0.456 | 29.6 | 12.64 | 132 |
| 122 | 117.1 | 74.1 | 5.13 | 0.348 | 21.8 | 12.12 | 106 |
| 123 | 144.75 | 62.25 | 4.82 | 0.188 | 45.5 | 13.7 | 185 |
| 124 | 190.5 | 21.9 | 28.1 | 0.381 | 190.5 | 24.05 | 481 |
| 125 | 165 | 27 | 23 | 0.33 | 165 | 21.5 | 430 |
| 126 | 190.5 | 51.9 | 9.05 | 0.181 | 90.5 | 15.62 | 281 |
| 127 | 118 | 42.8 | 13.6 | 0.236 | 118 | 17.44 | 336 |
| 128 | 105.5 | 47.8 | 11.1 | 0.211 | 105.5 | 16.44 | 311 |
| 129 | 175 | 25 | 25 | 0.35 | 175 | 22.5 | 450 |
| 130 | 197.5 | 50.5 | 9.75 | 0.195 | 97.5 | 15.9 | 295 |
| 131 | 110 | 46 | 12 | 0.22 | 110 | 16.8 | 320 |
| 132 | 151 | 59.8 | 5.1 | 0.102 | 51 | 14.04 | 202 |
| 133 | 198.5 | 20.3 | 29.7 | 0.397 | 198.5 | 24.85 | 497 |
| 134 | 127.95 | 69.45 | 5.2 | 0.496 | 31.1 | 12.74 | 137 |
| 135 | 118.5 | 42.6 | 13.7 | 0.237 | 118.5 | 17.48 | 337 |
| 136 | 138.5 | 34.6 | 17.7 | 0.277 | 138.5 | 19.08 | 377 |
| 137 | 133 | 36.8 | 16.6 | 0.266 | 133 | 18.64 | 366 |
| 138 | 149.65 | 60.15 | 5.45 | 0.0992 | 49.7 | 13.98 | 199 |
| 139 | 178 | 54.4 | 7.8 | 0.156 | 78 | 15.12 | 256 |
| 140 | 169.5 | 26.1 | 23.9 | 0.339 | 169.5 | 21.95 | 439 |
| 141 | 134.95 | 66.45 | 5.43 | 0.1656 | 37.1 | 13.14 | 157 |
| 142 | 193.5 | 21.3 | 28.7 | 0.387 | 193.5 | 24.35 | 487 |
| 143 | 141.25 | 63.75 | 5.32 | 0.08 | 42.5 | 13.5 | 175 |
| 144 | 102 | 49.2 | 10.4 | 0.204 | 102 | 16.16 | 304 |
| 145 | 171 | 55.8 | 7.1 | 0.142 | 71 | 14.84 | 242 |
| 146 | 143 | 63 | 4.8 | 0.184 | 44 | 13.6 | 180 |
| 147 | 185.5 | 52.9 | 8.55 | 0.171 | 85.5 | 15.42 | 271 |
| 148 | 129 | 38.4 | 15.8 | 0.258 | 129 | 18.32 | 358 |
| 149 | 193 | 51.4 | 9.3 | 0.186 | 93 | 15.72 | 286 |
| 150 | 182.5 | 23.5 | 26.5 | 0.365 | 182.5 | 23.25 | 465 |
| 151 | 179 | 54.2 | 7.9 | 0.158 | 79 | 15.16 | 258 |
| 152 | 168 | 26.4 | 23.6 | 0.336 | 168 | 21.8 | 436 |
| 153 | 185.5 | 22.9 | 27.1 | 0.371 | 185.5 | 23.55 | 471 |
| 154 | 100.5 | 49.8 | 10.1 | 0.201 | 100.5 | 16.04 | 301 |
| 155 | 145.8 | 61.8 | 5 | 0.0904 | 46.4 | 13.76 | 188 |
| 156 | 129.35 | 68.85 | 5 | 0.4528 | 32.3 | 12.82 | 141 |
| 157 | 189 | 22.2 | 27.8 | 0.378 | 189 | 23.9 | 478 |
| 158 | 180.5 | 53.9 | 8.05 | 0.161 | 80.5 | 15.22 | 261 |
| 159 | 160.5 | 27.9 | 22.1 | 0.321 | 160.5 | 21.05 | 421 |
| 160 | 124.45 | 70.95 | 5 | 0.1416 | 28.1 | 12.54 | 127 |
| 161 | 128.5 | 38.6 | 15.7 | 0.257 | 128.5 | 18.28 | 357 |
| 162 | 162.5 | 27.5 | 22.5 | 0.325 | 162.5 | 21.25 | 425 |
| 163 | 163.5 | 57.3 | 6.35 | 0.127 | 63.5 | 14.54 | 227 |
| 164 | 172 | 55.6 | 7.2 | 0.144 | 72 | 14.88 | 244 |
| 165 | 181.5 | 53.7 | 8.15 | 0.163 | 81.5 | 15.26 | 263 |
| 166 | 114.5 | 44.2 | 12.9 | 0.229 | 114.5 | 17.16 | 329 |
| 167 | 163.5 | 27.3 | 22.7 | 0.327 | 163.5 | 21.35 | 427 |
| 168 | 175 | 55 | 7.5 | 0.15 | 75 | 15 | 250 |
| 169 | 174 | 25.2 | 24.8 | 0.348 | 174 | 22.4 | 448 |
| 170 | 120.6 | 72.6 | 5 | 0.2328 | 24.8 | 12.32 | 116 |
| 171 | 172.5 | 25.5 | 24.5 | 0.345 | 172.5 | 22.25 | 445 |
| 172 | 119 | 42.4 | 13.8 | 0.238 | 119 | 17.52 | 338 |
| 173 | 198 | 20.4 | 29.6 | 0.396 | 198 | 24.8 | 496 |
| 174 | 186.5 | 22.7 | 27.3 | 0.373 | 186.5 | 23.65 | 473 |
| 175 | 111 | 45.6 | 12.2 | 0.222 | 111 | 16.88 | 322 |
| 176 | 165 | 57 | 6.5 | 0.13 | 65 | 14.6 | 230 |
| 177 | 165.5 | 26.9 | 23.1 | 0.331 | 165.5 | 21.55 | 431 |
| 178 | 158 | 28.4 | 21.6 | 0.316 | 158 | 20.8 | 416 |
| 179 | 109.5 | 46.2 | 11.9 | 0.219 | 109.5 | 16.76 | 319 |
| 180 | 196.5 | 50.7 | 9.65 | 0.193 | 96.5 | 15.86 | 293 |
| 181 | 129 | 69 | 5 | 0.052 | 32 | 12.8 | 140 |
| 182 | 113 | 44.8 | 12.6 | 0.226 | 113 | 17.04 | 326 |
| 183 | 159.5 | 58.1 | 5.95 | 0.119 | 59.5 | 14.38 | 219 |
| 184 | 131.1 | 68.1 | 5 | 0.2568 | 33.8 | 12.92 | 146 |
| 185 | 197 | 50.6 | 9.7 | 0.194 | 97 | 15.88 | 294 |
| 186 | 155 | 59 | 5.5 | 0.11 | 55 | 14.2 | 210 |
| 187 | 189.5 | 52.1 | 8.95 | 0.179 | 89.5 | 15.58 | 279 |
| 188 | 157.5 | 28.5 | 21.5 | 0.315 | 157.5 | 20.75 | 415 |
| 189 | 124.5 | 40.2 | 14.9 | 0.249 | 124.5 | 17.96 | 349 |
| 190 | 183 | 23.4 | 26.6 | 0.366 | 183 | 23.3 | 466 |
| 191 | 148.95 | 60.45 | 5 | 0.3976 | 49.1 | 13.94 | 197 |
| 192 | 106 | 47.6 | 11.2 | 0.212 | 106 | 16.48 | 312 |
| 193 | 149.5 | 30.2 | 19.9 | 0.299 | 149.5 | 19.96 | 399 |
| 194 | 142.65 | 63.15 | 5 | 0.3832 | 43.7 | 13.58 | 179 |
| 195 | 141.5 | 33.4 | 18.3 | 0.283 | 141.5 | 19.32 | 383 |
| 196 | 187 | 22.6 | 27.4 | 0.374 | 187 | 23.7 | 474 |
| 197 | 119.55 | 73.05 | 5 | 0.1304 | 23.9 | 12.26 | 113 |
| 198 | 196 | 50.8 | 9.6 | 0.192 | 96 | 15.84 | 292 |
| 199 | 123 | 40.8 | 14.6 | 0.246 | 123 | 17.84 | 346 |
| 200 | 127 | 39.2 | 15.4 | 0.254 | 127 | 18.16 | 354 |
| 201 | 177.5 | 54.5 | 7.75 | 0.155 | 77.5 | 15.1 | 255 |
| 202 | 169 | 26.2 | 23.8 | 0.338 | 169 | 21.9 | 438 |
| 203 | 130.75 | 68.25 | 5 | 0.056 | 33.5 | 12.9 | 145 |
| 204 | 147 | 31.2 | 19.4 | 0.294 | 147 | 19.76 | 394 |
| 205 | 177 | 54.6 | 7.7 | 0.154 | 77 | 15.08 | 254 |
| 206 | 136 | 35.6 | 17.2 | 0.272 | 136 | 18.88 | 372 |
| 207 | 134.25 | 66.75 | 5 | 0.064 | 36.5 | 13.1 | 155 |
| 208 | 141 | 33.6 | 18.2 | 0.282 | 141 | 19.28 | 382 |
| 209 | 149 | 30.4 | 19.8 | 0.298 | 149 | 19.92 | 398 |
| 210 | 136.7 | 65.7 | 5 | 0.2696 | 38.6 | 13.24 | 162 |
| 211 | 119.5 | 42.2 | 13.9 | 0.239 | 119.5 | 17.56 | 339 |
| 212 | 198 | 50.4 | 9.8 | 0.196 | 98 | 15.92 | 296 |
| 213 | 198.5 | 50.3 | 9.85 | 0.197 | 98.5 | 15.94 | 297 |
| 214 | 171.5 | 55.7 | 7.15 | 0.143 | 71.5 | 14.86 | 243 |
| 215 | 155.5 | 28.9 | 21.1 | 0.311 | 155.5 | 20.55 | 411 |
| 216 | 126 | 39.6 | 15.2 | 0.252 | 126 | 18.08 | 352 |
| 217 | 152 | 29.6 | 20.4 | 0.304 | 152 | 20.2 | 404 |
| 218 | 163 | 27.4 | 22.6 | 0.326 | 163 | 21.3 | 426 |
| 219 | 151.5 | 59.7 | 5.15 | 0.103 | 51.5 | 14.06 | 203 |
| 220 | 147.2 | 61.2 | 5 | 0.3936 | 47.6 | 13.84 | 192 |
| 221 | 101.5 | 49.4 | 10.3 | 0.203 | 101.5 | 16.12 | 303 |
| 222 | 158 | 58.4 | 5.8 | 0.116 | 58 | 14.32 | 216 |
| 223 | 176.5 | 24.7 | 25.3 | 0.353 | 176.5 | 22.65 | 453 |
| 224 | 125.5 | 39.8 | 15.1 | 0.251 | 125.5 | 18.04 | 351 |
| 225 | 117.45 | 73.95 | 5 | 0.1256 | 22.1 | 12.14 | 107 |
| 226 | 190 | 52 | 9 | 0.18 | 90 | 15.6 | 280 |
| 227 | 138.1 | 65.1 | 5 | 0.1728 | 39.8 | 13.32 | 166 |
| 228 | 173 | 25.4 | 24.6 | 0.346 | 173 | 22.3 | 446 |
| 229 | 144.05 | 62.55 | 5 | 0.2864 | 44.9 | 13.66 | 183 |
| 230 | 169 | 56.2 | 6.9 | 0.138 | 69 | 14.76 | 238 |
| 231 | 103.5 | 48.6 | 10.7 | 0.207 | 103.5 | 16.28 | 307 |
| 232 | 180.5 | 23.9 | 26.1 | 0.361 | 180.5 | 23.05 | 461 |
| 233 | 146.15 | 61.65 | 5 | 0.1912 | 46.7 | 13.78 | 189 |
| 234 | 148.6 | 60.6 | 5 | 0.0968 | 48.8 | 13.92 | 196 |
| 235 | 184 | 53.2 | 8.4 | 0.168 | 84 | 15.36 | 268 |
| 236 | 144.5 | 32.2 | 18.9 | 0.289 | 144.5 | 19.56 | 389 |
| 237 | 123.5 | 40.6 | 14.7 | 0.247 | 123.5 | 17.88 | 347 |
| 238 | 168.5 | 56.3 | 6.85 | 0.137 | 68.5 | 14.74 | 237 |
| 239 | 189.5 | 22.1 | 27.9 | 0.379 | 189.5 | 23.95 | 479 |
| 240 | 194 | 21.2 | 28.8 | 0.388 | 194 | 24.4 | 488 |
| 241 | 143.7 | 62.7 | 5 | 0.0856 | 44.6 | 13.64 | 182 |
| 242 | 135.3 | 66.3 | 5 | 0.1664 | 37.4 | 13.16 | 158 |
| 243 | 104.5 | 48.2 | 10.9 | 0.209 | 104.5 | 16.36 | 309 |
| 244 | 188 | 22.4 | 27.6 | 0.376 | 188 | 23.8 | 476 |
| 245 | 197 | 20.6 | 29.4 | 0.394 | 197 | 24.7 | 494 |
| 246 | 115 | 44 | 13 | 0.23 | 115 | 17.2 | 330 |
| 247 | 152.5 | 59.5 | 5.25 | 0.105 | 52.5 | 14.1 | 205 |
| 248 | 116 | 43.6 | 13.2 | 0.232 | 116 | 17.28 | 332 |
| 249 | 142.5 | 33 | 18.5 | 0.285 | 142.5 | 19.4 | 385 |
| 250 | 142.3 | 63.3 | 5 | 0.0824 | 43.4 | 13.56 | 178 |
| 251 | 191 | 21.8 | 28.2 | 0.382 | 191 | 24.1 | 482 |
| 252 | 143 | 32.8 | 18.6 | 0.286 | 143 | 19.44 | 386 |
| 253 | 170.5 | 55.9 | 7.05 | 0.141 | 70.5 | 14.82 | 241 |
| 254 | 104 | 48.4 | 10.8 | 0.208 | 104 | 16.32 | 308 |
| 255 | 127.5 | 39 | 15.5 | 0.255 | 127.5 | 18.2 | 355 |
| 256 | 193.5 | 51.3 | 9.35 | 0.187 | 93.5 | 15.74 | 287 |
| 257 | 164.5 | 27.1 | 22.9 | 0.329 | 164.5 | 21.45 | 429 |
| 258 | 116.4 | 74.4 | 5 | 0.1232 | 21.2 | 12.08 | 104 |
| 259 | FALSE | 50 | 10 | 0.2 | 100 | 16 | 300 |
| 260 | 185 | 23 | 27 | 0.37 | 185 | 23.5 | 470 |
| 261 | 131.45 | 67.95 | 5 | 0.1576 | 34.1 | 12.94 | 147 |
| 262 | 189 | 52.2 | 8.9 | 0.178 | 89 | 15.56 | 278 |
| 263 | 156.5 | 28.7 | 21.3 | 0.313 | 156.5 | 20.65 | 413 |
| 264 | 188 | 52.4 | 8.8 | 0.176 | 88 | 15.52 | 276 |
| 265 | 135.5 | 35.8 | 17.1 | 0.271 | 135.5 | 18.84 | 371 |
| 266 | 153.5 | 59.3 | 5.35 | 0.107 | 53.5 | 14.14 | 207 |
| 267 | 133.2 | 67.2 | 5 | 0.1616 | 35.6 | 13.04 | 152 |
| 268 | 124.8 | 70.8 | 5 | 0.2424 | 28.4 | 12.56 | 128 |
| 269 | 122 | 72 | 5 | 0.136 | 26 | 12.4 | 120 |
| 270 | 164 | 27.2 | 22.8 | 0.328 | 164 | 21.4 | 428 |
| 271 | 155 | 29 | 21 | 0.31 | 155 | 20.5 | 410 |
| 272 | 176 | 24.8 | 25.2 | 0.352 | 176 | 22.6 | 452 |
| 273 | 177.5 | 24.5 | 25.5 | 0.355 | 177.5 | 22.75 | 455 |
| 274 | 181.5 | 23.7 | 26.3 | 0.363 | 181.5 | 23.15 | 463 |
| 275 | 123.4 | 71.4 | 5 | 0.1392 | 27.2 | 12.48 | 124 |
| 276 | 128 | 38.8 | 15.6 | 0.256 | 128 | 18.24 | 356 |
| 277 | 132.85 | 67.35 | 5 | 0.1608 | 35.3 | 13.02 | 151 |
| 278 | 133.5 | 36.6 | 16.7 | 0.267 | 133.5 | 18.68 | 367 |
| 279 | 150.5 | 29.9 | 20.1 | 0.301 | 150.5 | 20.05 | 401 |
| 280 | 192 | 51.6 | 9.2 | 0.184 | 92 | 15.68 | 284 |
| 281 | 154 | 59.2 | 5.4 | 0.108 | 54 | 14.16 | 208 |
| 282 | 127.6 | 69.6 | 5 | 0.1488 | 30.8 | 12.72 | 136 |
| 283 | 177 | 24.6 | 25.4 | 0.354 | 177 | 22.7 | 454 |
| 284 | 139 | 34.4 | 17.8 | 0.278 | 139 | 19.12 | 378 |
| 285 | 137.05 | 65.55 | 5 | 0.2704 | 38.9 | 13.26 | 163 |
| 286 | 175.5 | 24.9 | 25.1 | 0.351 | 175.5 | 22.55 | 451 |
| 287 | 125.5 | 70.5 | 5 | 0.144 | 29 | 12.6 | 130 |
| 288 | 131.8 | 67.8 | 5 | 0.2584 | 34.4 | 12.96 | 148 |
| 289 | 116.5 | 43.4 | 13.3 | 0.233 | 116.5 | 17.32 | 333 |
| 290 | 148.5 | 30.6 | 19.7 | 0.297 | 148.5 | 19.88 | 397 |
| 291 | 137 | 35.2 | 17.4 | 0.274 | 137 | 18.96 | 374 |
| 292 | 134.5 | 36.2 | 16.9 | 0.269 | 134.5 | 18.76 | 369 |
| 293 | 154 | 29.2 | 20.8 | 0.308 | 154 | 20.4 | 408 |
| 294 | 193 | 21.4 | 28.6 | 0.386 | 193 | 24.3 | 486 |
| 295 | 122.5 | 41 | 14.5 | 0.245 | 122.5 | 17.8 | 345 |
| 296 | 188.5 | 52.3 | 8.85 | 0.177 | 88.5 | 15.54 | 277 |
| 297 | 171.5 | 25.7 | 24.3 | 0.343 | 171.5 | 22.15 | 443 |
| 298 | 124.1 | 71.1 | 5 | 0.1408 | 27.8 | 12.52 | 126 |
| 299 | 140.5 | 33.8 | 18.1 | 0.281 | 140.5 | 19.24 | 381 |
| 300 | 191 | 51.8 | 9.1 | 0.182 | 91 | 15.64 | 282 |
| 301 | 154.5 | 59.1 | 5.45 | 0.109 | 54.5 | 14.18 | 209 |
| 302 | 136 | 66 | 5 | 0.168 | 38 | 13.2 | 160 |
| 303 | 145.45 | 61.95 | 5 | 0.2896 | 46.1 | 13.74 | 187 |
| 304 | 132.15 | 67.65 | 5 | 0.1592 | 34.7 | 12.98 | 149 |
| 305 | 145.1 | 62.1 | 5 | 0.2888 | 45.8 | 13.72 | 186 |
| 306 | 123.75 | 71.25 | 5 | 0.14 | 27.5 | 12.5 | 125 |
| 307 | 129.7 | 68.7 | 5 | 0.1536 | 32.6 | 12.84 | 142 |
| 308 | 161.5 | 57.7 | 6.15 | 0.123 | 61.5 | 14.46 | 223 |
| 309 | 195.5 | 50.9 | 9.55 | 0.191 | 95.5 | 15.82 | 291 |
| 310 | 136.5 | 35.4 | 17.3 | 0.273 | 136.5 | 18.92 | 373 |
| 311 | 147.55 | 61.05 | 5 | 0.0944 | 47.9 | 13.86 | 193 |
| 312 | 194 | 51.2 | 9.4 | 0.188 | 94 | 15.76 | 288 |
| 313 | 180 | 54 | 8 | 0.16 | 80 | 15.2 | 260 |
| 314 | 121.3 | 72.3 | 5 | 0.1344 | 25.4 | 12.36 | 118 |
| 315 | 150.5 | 59.9 | 5.05 | 0.101 | 50.5 | 14.02 | 201 |
| 316 | 199.5 | 50.1 | 9.95 | 0.199 | 99.5 | 15.98 | 299 |
| 317 | 130.05 | 68.55 | 5 | 0.1544 | 32.9 | 12.86 | 143 |
| 318 | 131.5 | 37.4 | 16.3 | 0.263 | 131.5 | 18.52 | 363 |
| 319 | 164.5 | 57.1 | 6.45 | 0.129 | 64.5 | 14.58 | 229 |
| 320 | 119.9 | 72.9 | 5 | 0.1312 | 24.2 | 12.28 | 114 |
| 321 | 157 | 28.6 | 21.4 | 0.314 | 157 | 20.7 | 414 |
| 322 | 121 | 41.6 | 14.2 | 0.242 | 121 | 17.68 | 342 |
| 323 | 161.5 | 27.7 | 22.3 | 0.323 | 161.5 | 21.15 | 423 |
| 324 | 144 | 32.4 | 18.8 | 0.288 | 144 | 19.52 | 388 |
| 325 | 101 | 49.6 | 10.2 | 0.202 | 101 | 16.08 | 302 |
| 326 | 140.2 | 64.2 | 5 | 0.1776 | 41.6 | 13.44 | 172 |
| 327 | 115 | 75 | 5 | 0.02 | 20 | 12 | 100 |
| 328 | 162 | 57.6 | 6.2 | 0.124 | 62 | 14.48 | 224 |
| 329 | 153.5 | 29.3 | 20.7 | 0.307 | 153.5 | 20.35 | 407 |
| 330 | 199 | 50.2 | 9.9 | 0.198 | 99 | 15.96 | 298 |
| 331 | 196 | 20.8 | 29.2 | 0.392 | 196 | 24.6 | 492 |
| 332 | 153 | 29.4 | 20.6 | 0.306 | 153 | 20.3 | 406 |
| 333 | 186 | 52.8 | 8.6 | 0.172 | 86 | 15.44 | 272 |
| 334 | 127.25 | 69.75 | 5 | 0.148 | 30.5 | 12.7 | 135 |
| 335 | 192 | 21.6 | 28.4 | 0.384 | 192 | 24.2 | 484 |
| 336 | 158.5 | 28.3 | 21.7 | 0.317 | 158.5 | 20.85 | 417 |
| 337 | 157.5 | 58.5 | 5.75 | 0.115 | 57.5 | 14.3 | 215 |
| 338 | 143.35 | 62.85 | 5 | 0.2848 | 44.3 | 13.62 | 181 |
| 339 | 161 | 27.8 | 22.2 | 0.322 | 161 | 21.1 | 422 |
| 340 | 139.5 | 64.5 | 5 | 0.076 | 41 | 13.4 | 170 |
| 341 | 107 | 47.2 | 11.4 | 0.214 | 107 | 16.56 | 314 |
| 342 | 122.7 | 71.7 | 5 | 0.0376 | 26.6 | 12.44 | 122 |
| 343 | 170 | 56 | 7 | 0.14 | 70 | 14.8 | 240 |
| 344 | 184.5 | 23.1 | 26.9 | 0.369 | 184.5 | 23.45 | 469 |
| 345 | 143.5 | 32.6 | 18.7 | 0.287 | 143.5 | 19.48 | 387 |
| 346 | 162.5 | 57.5 | 6.25 | 0.125 | 62.5 | 14.5 | 225 |
| 347 | 168 | 56.4 | 6.8 | 0.136 | 68 | 14.72 | 236 |
| 348 | 103 | 48.8 | 10.6 | 0.206 | 103 | 16.24 | 306 |
| 349 | 195.5 | 20.9 | 29.1 | 0.391 | 195.5 | 24.55 | 491 |
| 350 | 181 | 23.8 | 26.2 | 0.362 | 181 | 23.1 | 462 |
| 351 | 150 | 32.8 | 6.54 | 0.374 | 149.52 | 19.6 | 164 |
| 352 | 178.5 | 25.7 | 5.43 | 0.785 | 157 | 23.55 | 257 |
| 353 | 176 | 25.2 | 5.48 | 0.76 | 152 | 22.8 | 252 |
| 354 | 150 | 20.2 | 7.485 | 0.1535 | 156 | 20.3 | 101 |
| 355 | 165.5 | 23.1 | 5.69 | 0.655 | 131 | 19.65 | 231 |
| 356 | 156.5 | 21.3 | 5.87 | 0.565 | 113 | 16.95 | 213 |
| 357 | 150 | 20.6 | 7.455 | 0.1605 | 168 | 20.9 | 103 |
| 358 | 150 | 23.4 | 7.245 | 0.2095 | 189 | 16.1 | 117 |
| 359 | 150 | 36.8 | 6.24 | 0.444 | 183.12 | 22.6 | 184 |
| 360 | 150 | 34.8 | 6.39 | 0.409 | 166.32 | 21.1 | 174 |
| 361 | 195 | 29 | 5.1 | 0.95 | 190 | 21.38 | 290 |
| 362 | 150 | 24.6 | 7.155 | 0.2305 | 161.28 | 17.27 | 123 |
| 363 | 150 | 38.8 | 6.09 | 0.479 | 142.8 | 24.1 | 194 |
| 364 | 183 | 26.6 | 5.34 | 0.83 | 166 | 18.68 | 266 |
| 365 | 176.5 | 25.3 | 5.47 | 0.765 | 153 | 22.95 | 253 |
| 366 | 150 | 27.6 | 6.93 | 0.283 | 151.2 | 20.2 | 138 |
| 367 | 183.5 | 26.7 | 5.33 | 0.835 | 167 | 25.05 | 267 |
| 368 | 150 | 22.4 | 7.32 | 0.192 | 166.5 | 23.6 | 112 |
| 369 | 150 | 21.6 | 7.38 | 0.178 | 198 | 22.4 | 108 |
| 370 | 150 | 33 | 6.525 | 0.3775 | 151.2 | 19.8 | 165 |
| 371 | 150 | 23.8 | 7.215 | 0.2165 | 198 | 16.5 | 119 |
| 372 | 150 | 31.2 | 6.66 | 0.346 | 136.08 | 23.6 | 156 |
| 373 | 160.5 | 22.1 | 5.79 | 0.605 | 121 | 18 | 221 |
| 374 | 150 | 21.8 | 7.365 | 0.1815 | 153 | 22.7 | 109 |

**Table S3**

**The verification data set**

| Sample number | Welding current **/A** | Arc voltage /V | Welding speed /(mm/s) | Wire elongation /(mm) | Inductance value/(H) | Welding gas flow /(L/min) | Residual stress  /(Mpa) |
| --- | --- | --- | --- | --- | --- | --- | --- |
| 1 | 161 | 57.8 | 6.1 | 0.122 | 61 | 14.44 | 222 |
| 2 | 149.3 | 60.3 | 4.8 | 0.0984 | 49.4 | 13.96 | 198 |
| 3 | 125.85 | 70.35 | 5.3 | 0.0448 | 29.3 | 12.62 | 131 |
| 4 | 122.35 | 71.85 | 5.21 | 0.368 | 26.3 | 12.42 | 121 |
| 5 | 146.85 | 61.35 | 5 | 0.0928 | 47.3 | 13.82 | 191 |
| 6 | 159 | 58.2 | 5.9 | 0.118 | 59 | 14.36 | 218 |
| 7 | 155.5 | 58.9 | 5.55 | 0.111 | 55.5 | 14.22 | 211 |
| 8 | 163 | 57.4 | 6.3 | 0.126 | 63 | 14.52 | 226 |
| 9 | 126.9 | 69.9 | 4.4 | 0.472 | 30.2 | 12.68 | 134 |
| 10 | 116.75 | 74.25 | 5.1 | 0.24 | 21.5 | 12.1 | 105 |
| 11 | 133.9 | 66.9 | 4.76 | 0.632 | 36.2 | 13.08 | 154 |
| 12 | 115.7 | 74.7 | 5 | 0.1216 | 20.6 | 12.04 | 102 |
| 13 | 173.5 | 55.3 | 7.35 | 0.147 | 73.5 | 14.94 | 247 |
| 14 | 182.5 | 53.5 | 8.25 | 0.165 | 82.5 | 15.3 | 265 |
| 15 | 138.45 | 64.95 | 5.1 | 0.536 | 40.1 | 13.34 | 167 |
| 16 | 120.25 | 72.75 | 4.84 | 0.032 | 24.5 | 12.3 | 115 |
| 17 | 169.5 | 56.1 | 6.95 | 0.139 | 69.5 | 14.78 | 239 |
| 18 | 167 | 56.6 | 6.7 | 0.134 | 67 | 14.68 | 234 |
| 19 | 138.8 | 64.8 | 5 | 0.744 | 40.4 | 13.36 | 168 |
| 20 | 157 | 58.6 | 5.7 | 0.114 | 57 | 14.28 | 214 |
| 21 | 118.5 | 73.5 | 5.22 | 0.028 | 23 | 12.2 | 110 |
| 22 | 150 | 60 | 5.14 | 0.1 | 50 | 14 | 200 |
| 23 | 133.55 | 67.05 | 4.8 | 0.624 | 35.9 | 13.06 | 153 |
| 24 | 152 | 59.6 | 5.2 | 0.104 | 52 | 14.08 | 204 |
| 25 | 130.4 | 68.4 | 4.92 | 0.352 | 33.2 | 12.88 | 144 |
| 26 | 141.6 | 63.6 | 5.7 | 0.208 | 42.8 | 13.52 | 176 |
| 27 | 135.65 | 66.15 | 5.5 | 0.372 | 37.7 | 13.18 | 159 |
| 28 | 156 | 58.8 | 5.6 | 0.112 | 56 | 14.24 | 212 |
| 29 | 140.55 | 64.05 | 4.9 | 0.484 | 41.9 | 13.46 | 173 |
| 30 | 141.95 | 63.45 | 4.65 | 0.316 | 43.1 | 13.54 | 177 |
| 31 | 139.85 | 64.35 | 6.2 | 0.468 | 41.3 | 13.42 | 171 |
| 32 | 136.35 | 65.85 | 5.7 | 0.288 | 38.3 | 13.22 | 161 |
| 33 | 139.15 | 64.65 | 5.6 | 0.352 | 40.7 | 13.38 | 169 |
| 34 | 153 | 59.4 | 5.3 | 0.106 | 53 | 14.12 | 206 |
| 35 | 166.5 | 56.7 | 6.65 | 0.133 | 66.5 | 14.66 | 233 |
| 36 | 132.5 | 67.5 | 5 | 0.16 | 35 | 13 | 150 |
| 37 | 126.2 | 70.2 | 4.76 | 0.456 | 29.6 | 12.64 | 132 |
| 38 | 117.1 | 74.1 | 5.13 | 0.348 | 21.8 | 12.12 | 106 |
| 39 | 144.75 | 62.25 | 4.82 | 0.188 | 45.5 | 13.7 | 185 |
| 40 | 127.95 | 69.45 | 5.2 | 0.496 | 31.1 | 12.74 | 137 |
| 41 | 149.65 | 60.15 | 5.45 | 0.0992 | 49.7 | 13.98 | 199 |
| 42 | 178 | 54.4 | 7.8 | 0.156 | 78 | 15.12 | 256 |
| 43 | 134.95 | 66.45 | 5.43 | 0.1656 | 37.1 | 13.14 | 157 |
| 44 | 141.25 | 63.75 | 5.32 | 0.08 | 42.5 | 13.5 | 175 |
| 45 | 143 | 63 | 4.8 | 0.184 | 44 | 13.6 | 180 |
| 46 | 145.8 | 61.8 | 5 | 0.0904 | 46.4 | 13.76 | 188 |
| 47 | 163.5 | 57.3 | 6.35 | 0.127 | 63.5 | 14.54 | 227 |
| 48 | 155 | 59 | 5.5 | 0.11 | 55 | 14.2 | 210 |
| 49 | 150 | 22.2 | 7.335 | 0.1885 | 162 | 23.3 | 111 |
| 50 | 158.5 | 21.7 | 5.83 | 0.585 | 117 | 18.7 | 217 |
| 51 | 150 | 32.8 | 6.54 | 0.374 | 149.52 | 19.6 | 164 |
| 52 | 178.5 | 25.7 | 5.43 | 0.785 | 157 | 23.55 | 257 |
| 53 | 176 | 25.2 | 5.48 | 0.76 | 152 | 22.8 | 252 |
| 54 | 150 | 20.2 | 7.485 | 0.1535 | 156 | 20.3 | 101 |
| 55 | 165.5 | 23.1 | 5.69 | 0.655 | 131 | 19.65 | 231 |
| 56 | 156.5 | 21.3 | 5.87 | 0.565 | 113 | 16.95 | 213 |
| 57 | 150 | 20.6 | 7.455 | 0.1605 | 168 | 20.9 | 103 |
| 58 | 150 | 23.4 | 7.245 | 0.2095 | 189 | 16.1 | 117 |
| 59 | 150 | 36.8 | 6.24 | 0.444 | 183.12 | 22.6 | 184 |
| 60 | 150 | 34.8 | 6.39 | 0.409 | 166.32 | 21.1 | 174 |
| 61 | 195 | 29 | 5.1 | 0.95 | 190 | 21.38 | 290 |
| 62 | 150 | 24.6 | 7.155 | 0.2305 | 161.28 | 17.27 | 123 |
| 63 | 150 | 38.8 | 6.09 | 0.479 | 142.8 | 24.1 | 194 |
| 64 | 183 | 26.6 | 5.34 | 0.83 | 166 | 18.68 | 266 |
| 65 | 176.5 | 25.3 | 5.47 | 0.765 | 153 | 22.95 | 253 |
| 66 | 150 | 27.6 | 6.93 | 0.283 | 151.2 | 20.2 | 138 |
| 67 | 183.5 | 26.7 | 5.33 | 0.835 | 167 | 25.05 | 267 |
| 68 | 150 | 22.4 | 7.32 | 0.192 | 166.5 | 23.6 | 112 |
| 69 | 150 | 21.6 | 7.38 | 0.178 | 198 | 22.4 | 108 |
| 70 | 150 | 33 | 6.525 | 0.3775 | 151.2 | 19.8 | 165 |
| 71 | 150 | 23.8 | 7.215 | 0.2165 | 198 | 16.5 | 119 |
| 72 | 150 | 31.2 | 6.66 | 0.346 | 136.08 | 23.6 | 156 |
| 73 | 160.5 | 22.1 | 5.79 | 0.605 | 121 | 18 | 221 |
| 74 | 150 | 21.8 | 7.365 | 0.1815 | 153 | 22.7 | 109 |

**Table S4**

**Predicted results and relative errors of welding verification samples**

| Sample number | Actual Residual stress | The predicted value of the APB algorithm | Analysis error |
| --- | --- | --- | --- |
| 1 | 222 | 232.6586548 | 4.80120% |
| 2 | 198 | 184.3617309 | 6.88801% |
| 3 | 131 | 124.1711304 | 5.21288% |
| 4 | 121 | 129.667395 | 7.16314% |
| 5 | 191 | 180.4715942 | 5.51225% |
| 6 | 218 | 234.529187 | 7.58220% |
| 7 | 211 | 222.0158325 | 5.22077% |
| 8 | 226 | 207.5712036 | 8.15433% |
| 9 | 134 | 126.2844116 | 5.75790% |
| 10 | 105 | 112.8535766 | 7.47960% |
| 11 | 154 | 141.4984253 | 8.11791% |
| 12 | 102 | 110.0386108 | 7.88099% |
| 13 | 247 | 230.6351196 | 6.62546% |
| 14 | 265 | 253.2728882 | 4.42533% |
| 15 | 167 | 181.6194214 | 8.75414% |
| 16 | 115 | 123.4832397 | 7.37673% |
| 17 | 239 | 225.4551391 | 5.66731% |
| 18 | 234 | 248.6829689 | 6.27477% |
| 19 | 168 | 177.4330374 | 5.61490% |
| 20 | 214 | 228.9185705 | 6.97129% |
| 21 | 110 | 119.1351006 | 8.30464% |
| 22 | 200 | 216.4136914 | 8.20685% |
| 23 | 153 | 162.8400967 | 6.43144% |
| 24 | 204 | 215.6966847 | 5.73367% |
| 25 | 144 | 137.3940622 | 4.58746% |
| 26 | 176 | 166.6286252 | 5.32464% |
| 27 | 159 | 148.5027904 | 6.60202% |
| 28 | 212 | 226.1145083 | 6.65779% |
| 29 | 173 | 163.9547512 | 5.22847% |
| 30 | 177 | 186.9410053 | 5.61639% |
| 31 | 171 | 180.7859741 | 5.72279% |
| 32 | 161 | 173.8766281 | 7.99791% |
| 33 | 169 | 158.2292862 | 6.37320% |
| 34 | 206 | 217.9072726 | 5.78023% |
| 35 | 233 | 222.9497364 | 4.31342% |
| 36 | 150 | 162.3952092 | 8.26347% |
| 37 | 132 | 122.9272156 | 6.87332% |
| 38 | 106 | 112.8223365 | 6.43617% |
| 39 | 185 | 174.1836982 | 5.84665% |
| 40 | 137 | 126.9678491 | 7.32274% |
| 41 | 199 | 212.8027268 | 6.93604% |
| 42 | 256 | 267.6247278 | 4.54091% |
| 43 | 157 | 145.5570687 | 7.28849% |
| 44 | 175 | 162.2699759 | 7.27430% |
| 45 | 180 | 166.173343 | 7.68148% |
| 46 | 188 | 178.2476628 | 5.18741% |
| 47 | 227 | 245.1774556 | 8.00769% |
| 48 | 210 | 221.7148216 | 5.57849% |
| 49 | 111 | 116.0278756 | 4.52961% |
| 50 | 217 | 206.8951399 | 4.65662% |
| 51 | 164 | 150.4864 | 8.24000% |
| 52 | 257 | 240.51602 | 6.41400% |
| 53 | 252 | 273.5014212 | 8.53231% |
| 54 | 101 | 93.27855 | 7.64500% |
| 55 | 231 | 211.507065 | 8.43850% |
| 56 | 213 | 197.1227201 | 7.45412% |
| 57 | 103 | 95.971074 | 6.82420% |
| 58 | 117 | 123.2761608 | 5.36424% |
| 59 | 184 | 172.1407584 | 6.44524% |
| 60 | 174 | 187.83996 | 7.95400% |
| 61 | 290 | 280.7438797 | 4.52962% |
| 62 | 123 | 130.6054236 | 4.65662% |
| 63 | 194 | 183.6831925 | 3.19177% |
| 64 | 266 | 277.8430084 | 6.18327% |
| 65 | 253 | 243.6645364 | 5.31794% |
| 66 | 138 | 130.1658734 | 4.45226% |
| 67 | 267 | 249.984624 | 6.37280% |
| 68 | 112 | 102.430384 | 8.54430% |
| 69 | 108 | 100.71648 | 6.74400% |
| 70 | 165 | 177.15126 | 7.36440% |
| 71 | 119 | 108.67794 | 8.67400% |
| 72 | 156 | 165.30852 | 5.96700% |
| 73 | 221 | 206.90462 | 6.37800% |
| 74 | 109 | 99.57804 | 8.64400% |
